# [Pemetrexed + Sorafenib] lethality is increased by inhibition of ERBB1/2/3-PI3K-NFκB compensatory survival signaling

**DOI:** 10.18632/oncotarget.8281

**Published:** 2016-03-22

**Authors:** Laurence Booth, Jane L. Roberts, Mehrad Tavallai, John Chuckalovcak, Daniel K. Stringer, Antonis E. Koromilas, David L. Boone, William P. McGuire, Andrew Poklepovic, Paul Dent

**Affiliations:** ^1^ Department of Biochemistry and Molecular Biology, Virginia Commonwealth University, Richmond, VA, USA; ^2^ Department of Medicine, Virginia Commonwealth University, Richmond, VA, USA; ^3^ Department of Bio-Rad Laboratories, Hercules, CA, USA; ^4^ Department of Microbiology and Immunology, Indiana University School of Medicine-South Bend, South Bend, IN, USA; ^5^ Department of Oncology, Lady Davis Institute for Medical Research, Montreal, QC, Canada

**Keywords:** pemetrexed, sorafenib, ERBB1, PTEN

## Abstract

In the completed phase I trial NCT01450384 combining the anti-folate pemetrexed and the multi-kinase inhibitor sorafenib it was observed that 20 of 33 patients had prolonged stable disease or tumor regression, with one complete response and multiple partial responses. The pre-clinical studies in this manuscript were designed to determine whether [pemetrexed + sorafenib] –induced cell killing could be rationally enhanced by additional signaling modulators. Multiplex assays performed on tumor material that survived and re-grew after [pemetrexed + sorafenib] exposure showed increased phosphorylation of ERBB1 and of NFκB and IκB; with reduced IκB and elevated G-CSF and KC protein levels. Inhibition of JAK1/2 downstream of the G-CSF/KC receptors did not enhance [pemetrexed + sorafenib] lethality whereas inhibition of ERBB1/2/4 using kinase inhibitory agents or siRNA knock down of ERBB1/2/3 strongly promoted killing. Inhibition of ERBB1/2/4 blocked [pemetrexed + sorafenib] stimulated NFκB activation and SOD2 expression; and expression of IκB S32A S36A significantly enhanced [pemetrexed + sorafenib] lethality. Sorafenib inhibited HSP90 and HSP70 chaperone ATPase activities and reduced the interactions of chaperones with clients including c-MYC, CDC37 and MCL-1. *In vivo*, a 5 day transient exposure of established mammary tumors to lapatinib or vandetanib significantly enhanced the anti-tumor effect of [pemetrexed + sorafenib], without any apparent normal tissue toxicities. Identical data to that in breast cancer were obtained in NSCLC tumors using the ERBB1/2/4 inhibitor afatinib. Our data argue that the combination of pemetrexed, sorafenib and an ERBB1/2/4 inhibitor should be explored in a new phase I trial in solid tumor patients.

## INTRODUCTION

The anti-folate drug pemetrexed (abbreviated in this manuscript as “PTX”) (Alimta^®^) was FDA-approved for the treatment of advanced and metastatic non-small cell lung cancer (NSCLC) in 2004. Pemetrexed was developed as an inhibitor of thymidylate synthase (TS), however based on its continued anti-proliferative effect on cells *in vitro* in the presence of exogenous thymidine, preventing the cytotoxic effects of TS inhibition, it became apparent that pemetrexed has at least one secondary target [1–4]. Subsequently, the folate-dependent enzyme, aminoimidazole-carboxamide ribonucleotide formyl-transferase (AICART), was shown to be a secondary target for pemetrexed [1, 2]. Inhibition of AICART increases ZMP levels, and elevated [ZMP] causes activation of AMP-activated protein kinase (AMPK) and downstream inhibition of mammalian target of rapamycin (mTOR) and activation of ULK-1 [1, 2, 5]. Inhibition of mTOR and activation of ULK-1 stimulates autophagy in part by reducing phosphorylation of ULK1 Serine 757 and by increasing phosphorylation of ULK-1 S317; thus activating the ULK-1 kinase to phosphorylate ATG13 S318, and enabling the association of additional ATG proteins required to initiate formation of the autophagosome [6–10].

Sorafenib and regorafenib are multi-kinase inhibitors approved for the treatment of liver and kidney, and colon cancers, respectively [11, and references therein]. Sorafenib was originally developed as an inhibitor of RAF-1 in the ERK1/2 pathway. The steady state (7 day) C_max_ for sorafenib is ~21 μM in plasma, with ~99% of the drug protein bound based on *in vitro* human serum binding assays; though it is known that the drug is also rapidly taken up into tissues, and in addition patient data from clinical trials would argue that a significant amount of the drug has to be bioavailable, at least in the low micro-molar range, in a tumor based on its single agent effects by decreasing both ERK1/2 phosphorylation and reducing MCL-1 protein expression in tumor cells that are not specifically oncogene addicted [12, 13]. Indeed, it has been shown that some sorafenib metabolites such as M2, M4 and M5 can have up to 10-fold greater activity than the parent drug [14–16]. Our prior data have tended to argue using several sorafenib + drug combinations that PDGFRβ is a major target of sorafenib for its interactions with other agents e.g. with histone deacetylase inhibitors [12, 13].

A major biological effect of sorafenib is the induction of an endoplasmic stress (ER) / unfolded protein response (UPR), with reduced expression of proteins that have short half-lives such as MCL-1 and BCL-XL [17, 18]. Reduced MCL-1 levels due to sorafenib exposure have been linked in many tumor types to increased levels of apoptosis. Studies by our group have also linked high dose single agent sorafenib exposure to an increase in the levels of autophagic markers including increased numbers of LC3-GFP intense staining vesicles and elevated expression of Beclin1 and ATG5; lower sorafenib concentrations only caused a modest transient alteration in autophagy flux [12, 13]. Other studies from our groups have shown that based on the sorafenib dose the induction of ER stress may be a “protective” or a “toxic” event in the cellular response to the drug [e.g. 19].

We recently reported at the 2015 ASCO meeting data from a completed phase I trial to determine the maximum safe doses of [pemetrexed + sorafenib] that can be administered to a heavily pre-treated cancer patient population (NCT01450384) [20]. A new phase II study specifically in HER2 negative ER/PR negative breast cancer has opened at Massey Cancer Center in the winter of 2016 (NCT02624700). Based on the early preliminary NCT01450384 phase I trial findings in 2014, the present pre-clinical studies were initiated to define in a rational manner the most efficacious “third agent” that could enhance [pemetrexed + sorafenib] lethality.

## RESULTS AND DISCUSSION

**Figure 1:**
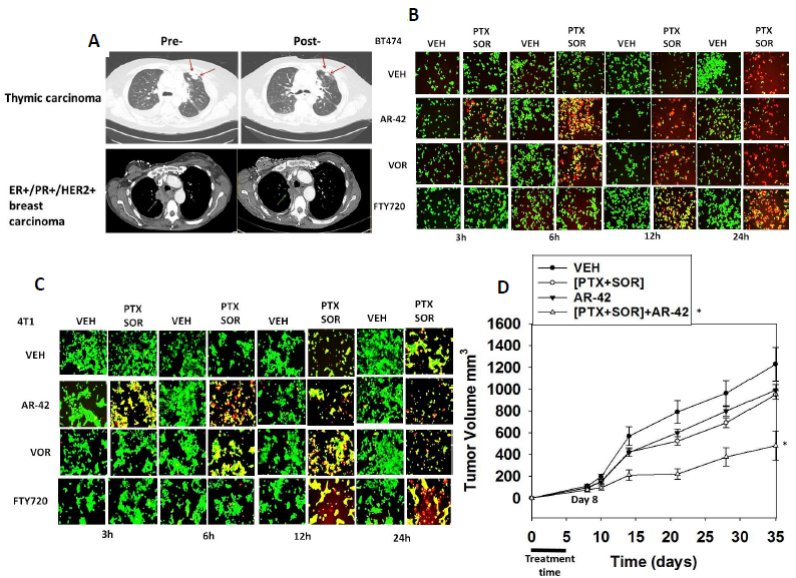
[Pemetrexed and Sorafenib] interact with modulators of bioactive lipid metabolism and with histone deacetylase inhibitors to kill tumor cells **A.** A phase I trial was performed combining pemetrexed and sorafenib at increasing doses (3 × 3 standard design). (see the companion Poklepovic et al manuscript, ref. 20). The data presented are from two patients on the trial: a thymic carcinoma patient and an ER+/PR+/HER2+ mammary carcinoma patient (see Methods of approach in reference 20 for additional details). **B.** BT474 human mammary carcinoma cells were treated with [pemetrexed (PTX, 0.5 μM) and sorafenib (2.0 μM)] in the presence or absence of the histone deacetylase inhibitors: FTY720 (0.1 μM); Vorinostat (0.5 μM); AR-42 (0.5 μM). Cells were examined 3-24h after treatment using a live / dead viability stain where green cells are viable and yellow / red cells are dead in WiScan Hermes instrument. **C.** 4T1 mouse mammary carcinoma cells were treated with [pemetrexed (PTX, 0.5 μM) and sorafenib (2.0 μM)] in the presence or absence of the histone deacetylase inhibitors: FTY720 (0.1 μM); Vorinostat (0.5 μM); AR-42 (0.5 μM). Cells were examined 3-24h after treatment using a live / dead viability stain where green cells are viable and yellow / red cells are dead in WiScan Hermes instrument. **D.** 4T1 cells (20,000) were injected into a BALB/c immune competent mouse fourth mammary fat pad. Five days after injection animals were randomly segregated into four groups. Animals were treated with vehicle (cremophore), [sorafenib (10 mg/kg) + pemetrexed (25 mg/kg)], AR-42 (10 mg/kg) or all three drugs simultaneously for 5 days QD. Please note the low level of sorafenib dosing in this specific animal study. Tumor volume was measured at Day 0 (no tumors evident) and then again beginning at Day 8 and then every seventh day and the actual increase in tumor volume calculated (2 studies, 8 animals total per group +/− SEM) *p < 0.05 lower than [sorafenib + pemetrexed] alone value.

As reported at the 2015 ASCO meeting, treatment of heavily pre-treated recurrent solid tumor patients with [pemetrexed + sorafenib] resulted in ~60% of all patients experiencing some degree of tumor growth delay (SD, PR, CR), with multiple partial responses and one complete response (Figure 1A; NCT01450384) [20].

As the two drug combination of [pemetrexed + sorafenib] effectively killed tumor cells *in vitro*, *in vivo* and in patients, we next searched for an efficacious translatable third drug which could be used to further enhance [pemetrexed + sorafenib] lethality. Multiple prior studies using sorafenib in the Dent laboratory have demonstrated that it synergizes with histone deacetylase inhibitors to kill tumor cells [12, 13]. Furthermore, data from our on-going phase I trial in hepatocellular carcinoma combining sorafenib and vorinostat has shown that multiple patients have displayed prolonged > 6 months stable disease (NCT01075113) (Poklepovic and Dent, unpublished observations). In the “triple negative” mouse mammary tumor cell line 4T1 [pemetrexed + sorafenib] interacted to kill, an effect that was enhanced by the histone deacetylase inhibitor and also inhibitor of sphingosine-1-phosphate signaling FTY720 (Fingolimod, Gilenya^®^) (Figure 1B). The ability of other clinically relevant histone deacetylase inhibitor drugs to enhance [pemetrexed + sorafenib] toxicity was also examined, and it was noted that the class I / class II histone deacetylase inhibitors vorinostat and AR-42 both strongly and more rapidly enhanced [sorafenib + pemetrexed] lethality than FTY720 (Figures 1B and 1C). Transient treatment of animals with low doses of [pemetrexed + sorafenib] modestly reduced the growth of established orthotopic 4T1 mouse mammary tumors in their syngeneic host BALB/c mouse (Figure 1D). Combined treatment of animals with [sorafenib + pemetrexed + AR-42] significantly reduced tumor growth below that of [pemetrexed + sorafenib] alone and enhanced animal survival using this highly aggressive ERBB1 expressing triple negative mammary carcinoma (*p* < 0.05).

**Figure 2:**
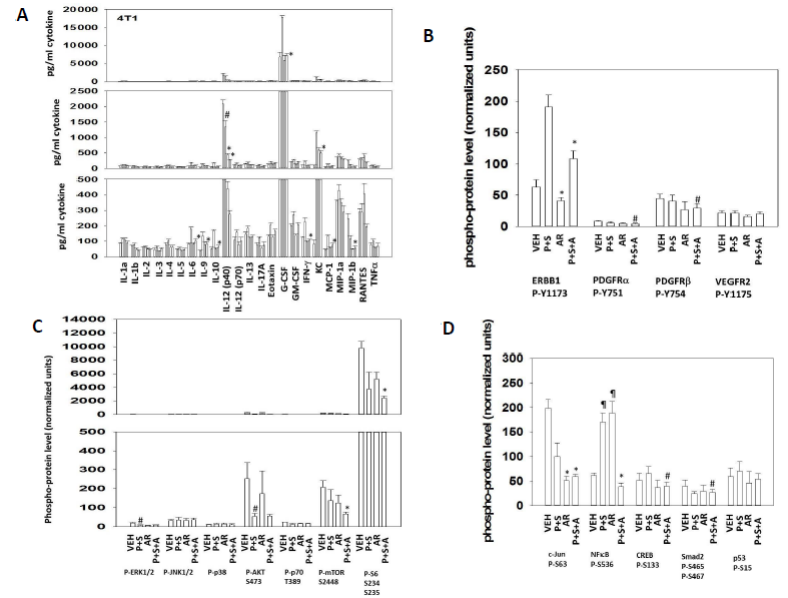
Multiplex data on plasma cytokine levels and tumor signal transduction protein phosphorylation after 7 days of mouse drug treatment **A.**-**D.** Animals carrying 4T1 tumors, five days after injection, were subjected to drug treatment for 5 days as per Figure 1D. After seven days, animals were sacrificed and blood and tumor obtained. Clarified plasma and tumor cell lysates were then subjected to multiplex assays as described in the Methods to detect the plasma levels of the indicated cytokines and phosphorylation status of signal transduction proteins using a Bio-Rad MAGPIX multiplex instrument (total 8 animals per condition, +/− SEM). *In Panels A-D the data are grouped in sets of four bars for each protein assessment, in order: vehicle treatment; [PTX+SOR] treatment; AR-42 treatment; [PTX+SOR] + AR-42 treatment.* # *p* < 0.05 less than vehicle control value; * *p* < 0.05 less than corresponding value without AR-42.

In parallel studies with the animals being exposed to drugs for 5 days and animals humanely sacrificed at day 7, mouse blood and tumors were analyzed for mouse cytokine levels with and without drug treatments using a Bio-Rad MAGPIX multiplex system and antibody micro-arrays (Figures 2A-2D). We noted that the expression of G-CSF was increased by [pemetrexed + sorafenib] treatment, which was reduced by AR-42. Basal expression of IL-12 (p40) and of KC (CXCL1) was high, that was reduced by AR-42 treatment. Similar findings were also obtained, albeit with lower cytokine levels, examining IL-6, IL-9, IL-10 and IFN-γ. [Pemetrexed + sorafenib] treatment increased the phosphorylation of ERBB1, which was reduced by AR-42 (Figure 2B). Similar more modest findings were noted for PDGFRα/β. This reduction in ERBB1 phosphorylation by co-exposure with AR-42 correlated with reduced phosphorylation of ERK1/2, AKT, p70 S6K and mTOR, which was also associated with reduced S6 phosphorylation (Figure 2C). Downstream of the membrane-to-nucleus signaling pathways, AR-42 reduced the levels of c-Jun phosphorylation and both [pemetrexed + sorafenib] as well as AR-42 increased the phosphorylation of the NFκB p65 subunit but when combined as [pemetrexed + sorafenib + AR-42] decreased NFκB p65 phosphorylation (Figure 2D). Collectively, the data in Figures 1 and 2 argues that the combination of [pemetrexed + sorafenib] with clinically relevant HDAC inhibitors represents a promising approach for the treatment of triple negative breast cancer.

**Figure 3:**
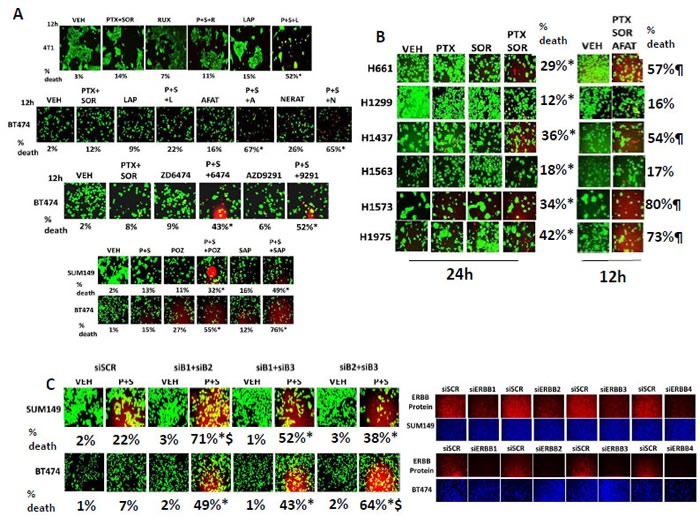
Inhibition of ERBB1/2 but not JAK1/2 significantly enhances tumor cell killing by [Pemetrexed and Sorafenib] **A.** BT474 and 4T1 cells were treated with vehicle control, [pemetrexed (0.5 μM) and sorafenib (2 μM)], ruxolitinib (0.5 μM), lapatinib (1 μM), afatinib (0.5 μM), neratinib (0.5 μM), ZD6474/Vandetanib (0.2 μM), sapitinib (0.2 μM), poziotinib (0.2 μM), AZD9291 (0.2 μM) or the drugs in combination as indicated. After 12h of incubation, cells were treated with live / dead reagent and cells examined using a Hermes WiScan microscope where red/yellow cells = dead; green cells = alive (*n* = 3 +/− SEM). * *p* < 0.05 greater than [PTX+SOR] treatment alone. **B.** NSCLC lines were treated with vehicle control, pemetrexed (0.5 μM) and/or sorafenib (2 μM) for 24h after which cell viability was determined by live/dead assay (+/− SEM) *p < 0.05 greater than pemetrexed alone value). In parallel studies NSCLC lines were treated with vehicle control, [pemetrexed (0.5 μM) and sorafenib (2 μM)], afatinib (0.5 μM), lapatinib (1.0 μM) or the drugs in combination. Twelve h after treatment cell viability was determined by live/dead assay (+/− SEM) ¶ *p* < 0.05 greater than corresponding 24h value of [pemetrexed + sorafenib] only. **C.** BT474 and SUM149 human mammary carcinoma cells were transfected with a scrambled siRNA (siSCR) or to knock down the expression of ERBB1, ERBB2, ERBB3, ERBB4 in the combinations indicated in the Figure. Twenty four h after transfection cells were treated with vehicle control or with [pemetrexed (0.5 μM) and sorafenib (2 μM)] as indicated. After 12h of incubation, cells were treated with live / dead reagent and cells examined using a Hermes WiScan microscope where red/yellow cells = dead; green cells = alive (+/− SEM) * *p* < 0.05 greater than P+S value in siSCR transfected cells; $ *p* < 0.05 greater than the other receptor combination values.

Based on the findings in Figure 2 and the identified molecular response biomarkers, we determined whether an inhibitor of KC and G-CSF signaling (i.e. the clinically relevant JAK1/2 inhibitor ruxolitinib), or an inhibitor of ERBB1/2/4 signaling (i.e. the clinically relevant inhibitor lapatinib) could modify the toxicity of [pemetrexed + sorafenib] in 4T1 and in BT474 cells. In 4T1 cells inhibition of ERBB1/2/4 signaling by use of lapatinib, but not inhibition of JAK1/2 signaling using ruxolitinib, enhanced [pemetrexed + sorafenib] lethality (Figure 3A, upper). Similar data were obtained in BT474 cells using the ERBB1/2/4 inhibitors afatinib, vandetanib, the recently approved 3^rd^ generation inhibitor AZD9291, sapitinib, poziotinib and neratinib, but not with either of the ERBB1 only specific inhibitor drugs gefitinib or erlotinib (Figure 3A, middle/lower panels).

The drugs afatinib and pemetrexed are approved for use in non-small cell lung cancer patients and we next determined the killing abilities of sorafenib, pemetrexed and afatinib in NSCLC cell lines. Sorafenib and pemetrexed interacted to kill with varying efficacies all of NSCLC lines tested; an effect that was enhanced by afatinib, regardless of whether the cell line expressed a mutant active ERBB1 or a mutant active N-RAS / K-RAS or lacked p16 / p53 function (Figure 3B). Molecular knock down of ERBB receptors recapitulated the effects caused by ERBB1/2/4 inhibitors in SUM149 and BT474 breast cancer cells with combined knock down of ERBB2 and ERBB3 facilitating strong killing in SUM149 cells and with combined knock down of ERBB1 and ERBB2 facilitating strong killing in BT474 cells (Figure 3C).

**Figure 4:**
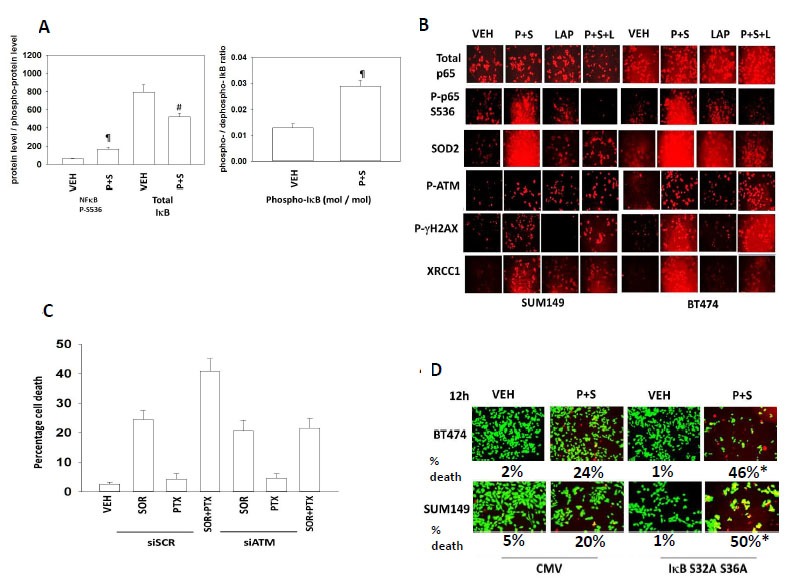
Inhibition of NFκB signaling prevents induction of SOD2 expression and enhances tumor cell killing by [Pemetrexed and Sorafenib]; [Pemetrexed + Sorafenib] causes a prolonged activation of ATM which promotes tumor cell killing **A.** Animals carrying 4T1 tumors, five days after injection, were subjected to drug treatment for 5 days as per Figure 1D. After seven days, animals were sacrificed and blood and tumor obtained. Clarified plasma and tumor cell lysates were then subjected to multiplex assays as described in the Methods to detect the plasma levels of the indicated cytokines and phosphorylation status of signal transduction proteins using a Bio-Rad MAGPIX multiplex instrument (total 8 animals per condition, +/− SEM). # *p* < 0.05 less than vehicle control value; ¶ *p* < 0.05 greater than vehicle control. **B.** BT474 and SUM149 cells were treated with vehicle control, [pemetrexed (0.5 μM) and sorafenib (2 μM)], lapatinib (1 μM), or the drugs in combination as indicated. Six h after drug treatment cells were fixed and permeabilized *in situ* and immuno-fluorescence was performed to determine the expression and the phosphorylation of the indicated proteins. **C.** *Lower graph*: BT474 mammary carcinoma cells were transfected with a scrambled siRNA (siSCR) or to knock down the expression of ATM. Twenty four h after transfection cells were treated with vehicle control or with pemetrexed (0.5 μM) and/or sorafenib (2 μM), as indicated. Twenty four h after treatment cells were isolated and viability determined by trypan blue inclusion (*n* = 3 +/− SEM). super-repressor protein IκB S32A S36A. Twenty four h after transfection cells were treated with vehicle control or [pemetrexed (0.5 μM) and sorafenib (2 μM)], as indicated. Twelve h after drug treatment cells were treated with live / dead reagent and cells examined using a Hermes WiScan microscope where red/yellow cells = dead; green cells = alive (+/− SEM) * *p* < 0.05 greater than corresponding value in CMV transfected cells.

Pemetrexed causes DNA damage and we next investigated the role of NFκB and ATM / DNA repair in survival signaling *in vitro* (Figure 4). [Pemetrexed + sorafenib] treatment not only resulted in surviving cells having increased phosphorylation of p65 NFκB but also with decreased total expression of IκB and increased the mole/mole phosphate ratio in IκB in tumor cells isolated after animal treatment (Figure 4A). As judged by immuno-fluorescent *in situ* staining, treatment of BT474 and SUM149 cells *in vitro* with [pemetrexed + sorafenib] increased the phosphorylation of p65 NFκB S536, indicative of increased transcriptional activity (Figure 4B). Lapatinib did not significantly alter basal p65 phosphorylation but abolished stimulated p65 phosphorylation when combined with [pemetrexed + sorafenib]. Mn Cu superoxide dismutase 2 (SOD2) is one well-recognized cyto-protective NFκB target gene and in agreement with our p65 phosphorylation data, lapatinib prevented [pemetrexed + sorafenib] -induced SOD2 expression. Our drug treatments that included pemetrexed caused increased phosphorylation of ATM and γH2AX, effects that were *not* modulated by lapatinib (Figure 4B). As DNA damage was occurring but was not being increased by lapatinib, we determined whether DNA repair genes whose activity can be stimulated by NFκB were being modulated. Treatment of cells with [pemetrexed + sorafenib] increased the expression of the NFκB target gene XRCC1, which was prevented by co-incubation with lapatinib. Knock down of ATM expression prevented the toxic interaction between sorafenib and pemetrexed (Figure 4C). Of note, the drug combination of [pemetrexed + sorafenib] activated the AMP-dependent kinase (AMPK, T172 phosphorylation) earlier at 6h and 12h than the responses observed for single drug exposure to either sorafenib or pemetrexed (data not shown). These findings suggest it is the persistence of unrepaired DNA damage, rather than increased DNA damage, that may be a portion of the mechanism by which ERBB1/2/4 inhibitors enhance [pemetrexed + sorafenib] lethality. Finally, we expressed the super-repressor protein IκB S32A S36A to block NFκB signaling. Expression of IκB S32A S36A significantly enhanced [pemetrexed + sorafenib] lethality (Figure 4D).

**Figure 5:**
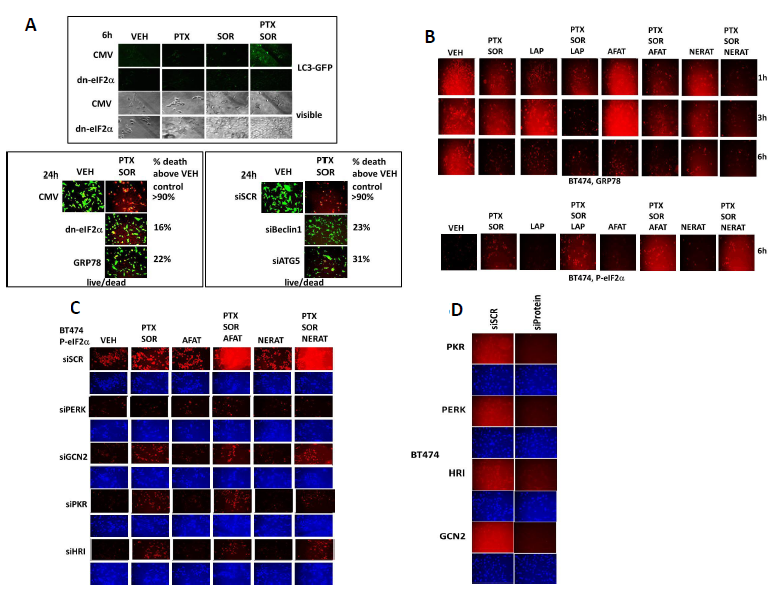
[Pemetrexed + Sorafenib] killing and its enhancement by ERBB1/2 inhibition relies on elevated endoplasmic reticulum stress and AMPK signaling **A.** *Upper*: SUM149 cells were transfected with a plasmid to express LC3-GFP in parallel with either an empty vector plasmid (CMV) or a plasmid to express dominant negative eIF2α S51A. Twenty four h after transfection cells were treated with vehicle control or with pemetrexed (0.5 μM) and / or sorafenib (2 μM) for 6h. Cells were imaged (10X) under fluorescent light (FITC channel) and afterwards under visible light. *Lower left*: SUM149 cells were transfected with either an empty vector plasmid (CMV) or a plasmid to express dominant negative eIF2α S51A or a plasmid to express GRP78 / BiP / HSPA5. *Lower right*: SUM149 cells were transfected with either a control scrambled siRNA (siSCR) or siRNA molecules to knock down the expression of Beclin1 or ATG5. Twenty four h after transfection cells were treated with vehicle control or with pemetrexed (0.5 μM) and sorafenib (2 μM) for 24h. Floating cells were re-attached to the plate by centrifugation and cell viability determined using a live / dead assay where viable cells stain green, and non-viable cells stain yellow and red (*n* = 3, +/− SEM). The effects of eIF2α S51A, GRP78, siBeclin1 and siATG5 on reducing cell killing were significant (*p* < 0.05). **B.** BT474 cells were treated with vehicle control, [pemetrexed (0.5 μM) and sorafenib (2 μM)], Lapatinib (1 μM), Afatinib (0.5 μM), Neratinib (0.5 μM) or the drugs in combination as indicated. Cells were fixed and permeabilized *in situ* 1-6h after drug treatment as indicated. Immuno-fluorescence was performed to determine the expression of GRP78 and the S51 phosphorylation of eIF2α. **C.** BT474 cells were transfected with a scrambled siRNA or with siRNA molecules to knock down the expression of PERK, GCN2, PKR or HRI. Twenty four h after transfection cells were treated with vehicle control, [pemetrexed (0.5 μM) and sorafenib (2 μM)], afatinib (0.5 μM), Neratinib (0.5 μM) or the drugs in combination as indicated. Six h after drug treatment cells were fixed and permeabilized *in situ* and immuno-fluorescence was performed to determine the S51 phosphorylation of eIF2α. **D.** Control immuno-fluorescence / in-situ western assays to show the expression levels of proteins after siRNA knock down.

Our prior studies demonstrated that endoplasmic reticulum stress signaling and a toxic form of autophagosome formation and autophagy flux with release of lysosomal proteases was a key mechanism by which [pemetrexed + sorafenib] killed tumor cells [6]. Treatment of cells with [pemetrexed + sorafenib] increased numbers of intense punctate LC3-GFP staining autophagosomes in cells in an eIF2α -dependent mechanism (Figure 5A, upper). Expression of dominant negative eIF2α S51A, over-expression of GRP78 or knock down of Beclin1 or ATG5 protected cells from [pemetrexed + sorafenib] lethality (Figure 5A, lower). Treatment of BT474 cells with [pemetrexed + sorafenib] reduced expression of the endoplasmic reticulum chaperone GRP78 (Figure 5B, upper). Notably the combination of [pemetrexed + sorafenib] with an ERBB1/2/4 inhibitor more rapidly promoted the reduction in GRP78 expression compared to the individual drugs. The reductions in GRP78 expression correlated with increased phosphorylation of the endoplasmic reticulum stress mediator eIF2α (Figure 5B, lower). Multiple kinases phosphorylate eIF2α, and we discovered that knock down of PKR-like endoplasmic reticulum kinase (PERK) most effectively suppressed the phosphorylation of eIF2α caused by [pemetrexed + sorafenib] and by the combination of an ERBB1/2/4 inhibitor with [pemetrexed + sorafenib] (Figure 5C). See also Figure 5D for control images of protein expression and molecular knock down effects.

**Figure 6:**
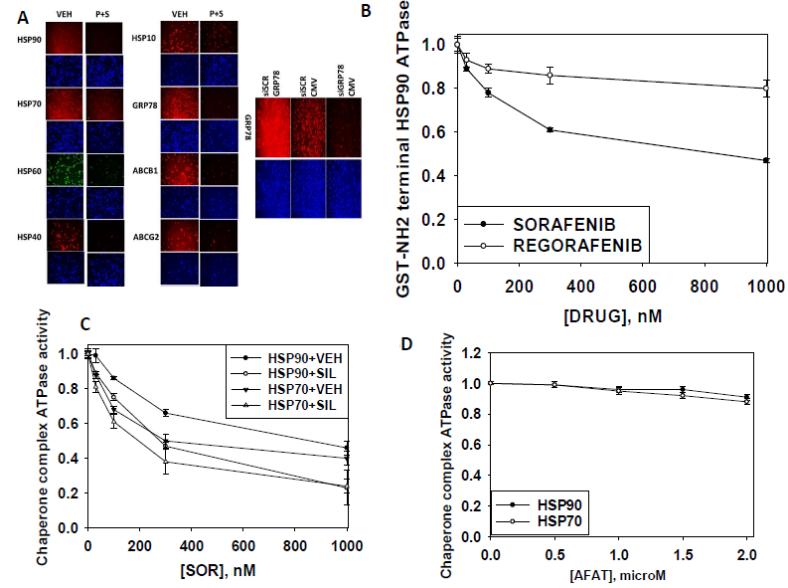
[Pemetrexed + Sorafenib] treatment regulates the expression of multiple chaperone proteins and plasma membrane drug efflux pumps **A.** SUM149 cells were treated with vehicle control or [pemetrexed (0.5 μM) and sorafenib (2 μM)] for 6h. Six h after drug treatment cells were fixed and permeabilized *in situ* and immuno-fluorescence was performed to determine the expression of chaperone proteins and drug efflux pumps. **B.** A GST-HSP90 NH2-terminal fragment containing the ATP binding domain of the chaperone was synthesized in *E. coli* and purified from other bacterial proteins using glutathione sepharose. The GST-HSP90 NH2-terminal fragment protein *was not* eluted off the sepharose beads. Equal portions of beads were immediately aliquoted into individual wells in a 96 well plate. Beads were resuspended in kinase reaction buffer containing vehicle control; sorafenib tosylate; regorafenib; (30 nM; 100 nM; 300 nM; 1 μM) in triplicate, and incubated for 30 min at 37°C. The reaction was started by addition of ATP-lite substrate. The plate was removed from the incubator and placed into a Vector 3 plate reader to determine the luminescence of the reactions under each treatment condition (*n* = 3 (x 3) +/− SEM). **C.** and **D.** GBM12 cells were transfected with a plasmid to express HSP70-GFP or to express FLAG-tagged HSP90. Twenty four h after transfection cells were treated with vehicle control or sildenafil (2 μM) for 1h. Chaperone proteins were immuno-precipitated using their tags in the presence of phosphatase inhibitors. Equal portions of precipitate sepharose beads were immediately aliquoted into individual wells in a 96 well plate. Beads were resuspended in ATPase reaction buffer containing vehicle control; sorafenib tosylate; afatinib; (30 nM; 100 nM; 300 nM; 1 μM; 2 μM) in triplicate, and incubated for 30 min at 37°C. The reaction was started by addition of ATP-lite substrate. The plate was removed from the incubator and placed into a Vector 3 plate reader to determine the luminescence of the reactions under each treatment condition (*n* = 3 (x 3) +/− SEM).

In multiple studies over the past decade we have shown that sorafenib can increase endoplasmic reticulum stress signaling in part by reducing the expression of chaperone proteins as well as expression of drug efflux pumps [17, 18, 21–24]. Treatment of breast cancer cells with [pemetrexed + sorafenib] reduced the immuno-fluorescence detection of the chaperone proteins HSP90, GRP78, HSP70, HSP60, HSP40, HSP27, HSP10 as well as the multi-drug efflux pumps ABCB1 and ABCG2 (Figure 6A). Sorafenib, but not regorafenib or afatinib, inhibited the ATPase activities of bacterial synthesized HSP90 and eukaryotic cell synthesized HSP90 and eukaryotic cell synthesized HSP70 (Figures 6B-6D). Treatment of cells for 60 min with the phosphodiesterase 5 inhibitor sildenafil (Viagra) enhanced the ability of sorafenib to inhibit chaperone ATPase activities *in vitro*; the chaperones having been isolated from eukaryotic cells by immuno-precipitation.

**Figure 7:**
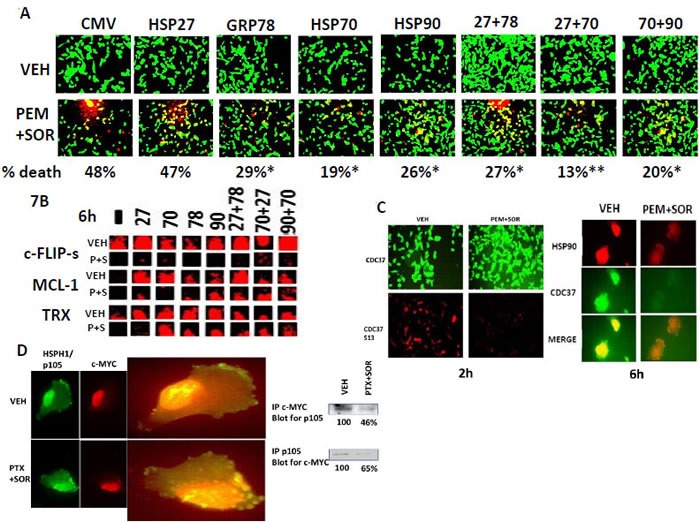
[Pemetrexed + sorafenib] lethality is reduced by over-expression of GRP78, HSP70 and HSP90 **A.** GBM12 cells were transfected with empty vector control (CMV) or with plasmids to express HSP27, GRP78, HSP70 or HSP90, alone or in combination as indicated. Twenty four h after transfection cells were treated with vehicle control or with [pemetrexed (1.0 μM) + sorafenib (2.0 μM)] for 24h. Cell viability determined using a live / dead assay where viable cells stain green, and non-viable cells stain yellow and red (*n* = 3, +/− SEM) * *p* < 0.05 less than corresponding CMV value; **p < 0.05 less than value obtained in single transfection to express HSP70. **B.** GBM12 cells were transfected with empty vector control (CMV) or with plasmids to express HSP27, GRP78, HSP70 or HSP90, alone or in combination as indicated. Twenty four h after transfection cells were treated with vehicle control or with [pemetrexed (1.0 μM) + sorafenib (2.0 μM)] for 6h. Six h after drug treatment cells were fixed and permeabilized *in situ* and immuno-fluorescence was performed to determine the expression of the proteins c-FLIP-s, MCL-1 or thioredoxin (TRX). **C.** *Left panels*: GBM12 cells were treated for 2h with [pemetrexed (1.0 μM) and sorafenib (2.0 μM)] after which cells were fixed in place and permeabilized using 0.5% Triton X100. Immuno-fluorescence was performed to detect the expression levels of CDC37 and the phosphorylation of CDC37 S13 at 10X magnification. *Right panels*: GBM12 cells were treated with vehicle, [pemetrexed (>1.0 μM) + sorafenib (2.0 μM)] for 6h after which cells were fixed in place and permeabilized using 0.5% Triton X100. Immuno-fluorescence was performed to detect the co-localization of CDC37 and HSP90 at 60X magnification. **D.** *Left:* BT474 and SUM149 cells were treated for 2h with vehicle or with [pemetrexed (0.5 μM) and sorafenib (2 μM)]. Two h after drug treatment cells were fixed and permeabilized *in situ* and immuno-fluorescence was performed to determine the expression of the HSPH1 / p105 chaperone protein and c-MYC protein at 10X magnification, and the co-localization of p105 and c-MYC at 60X magnification. *Right:* BT474 and SUM149 cells were treated for 6h with vehicle control or with [pemetrexed (0.5 μM) and sorafenib (2 μM)]. Six h after drug treatment cells were lysed and either p105 or c-MYC immuno-precipitated. Immuno-precipitates of p105 were subjected to SDS PAGE and immuno-blotting performed to determine the expression / association of c-MYC. Immuno-precipitates of c-MYC were subjected to SDS PAGE and immuno-blotting performed to determine the expression / association of p105.

Over-expression of GRP78, HSP70 or HSP90 significantly reduced the lethality of [pemetrexed + sorafenib] (Figure 7A). Although over-expression of HSP27 did not protect cells from [pemetrexed + sorafenib] expression of HSP27 in the presence of HSP70, HSP27 did enhance the cyto-protective effect of HSP70. Downstream of the chaperones, over-expression of [HSP27 + GRP78]; [HSP27 + HSP70]; and [HSP70 + HSP90] enhanced the basal expression of thioredoxin (TRX), MCL-1 and c-FLIP-s, and diminished the ability of [pemetrexed + sorafenib] to reduce their protein expression (Figure 7B). HSP90 function is controlled in part by its essential co-chaperone CDC37. CDC37, when phosphorylated on Serine 13 by casein kinase 2, facilitates HSP90 chaperoning of cyclin dependent kinases, IκB kinases and eIF2α kinases. Serine 13 phosphorylation is thought to stabilize CDC37 into a more compact conformation with enhanced secondary structure. A 2h exposure of cells to [pemetrexed + sorafenib] reduced the phosphorylation of CDC37 Serine 13 without any apparent change in the total expression of CDC37 itself at that 2h time point (Figure 7C, left panels). In co-localization studies (i.e. red + green = yellow), we noted that at the 6h incubation time point [pemetrexed + sorafenib] treatment had reduced the total expression of both HSP90 and of CDC37 themselves and decreased the amount of CDC37-HSP90 co-localization (i.e. reduced yellow staining; increased red-orange staining) (Figure 7C, right panels). The oncogenic transcription factor c-MYC is known to be chaperoned in part by the chaperones HSPH1/p105, HSP60 and HSP27. In agreement with the drug combination causing chaperone dysfunction, treatment of tumor cells with [pemetrexed + sorafenib] reduced c-MYC - p105 co-localization in the nucleus without apparently altering the total amount of nuclear localized c-MYC protein (Figure 7D). We also observed increased numbers of vesicles in the cytosol of [pemetrexed + sorafenib] treated cells that contained high levels of HSPH1/p105 but very low levels of c-MYC. In agreement with the apparent reduced association in the nucleus between c-MYC and HSPH1/p105, the amount of HSPH1/p105 co-immuno-precipitating with c-MYC and the amount of c-MYC co-immuno-precipitating with HSPH1/p105 were both reduced by ~50% following [pemetrexed + sorafenib] exposure (Figure 7D, blots to the right). Thus [pemetrexed + sorafenib] treatment by inhibiting the function of HSPH1/p105, also reduces c-MYC function.

**Figure 8:**
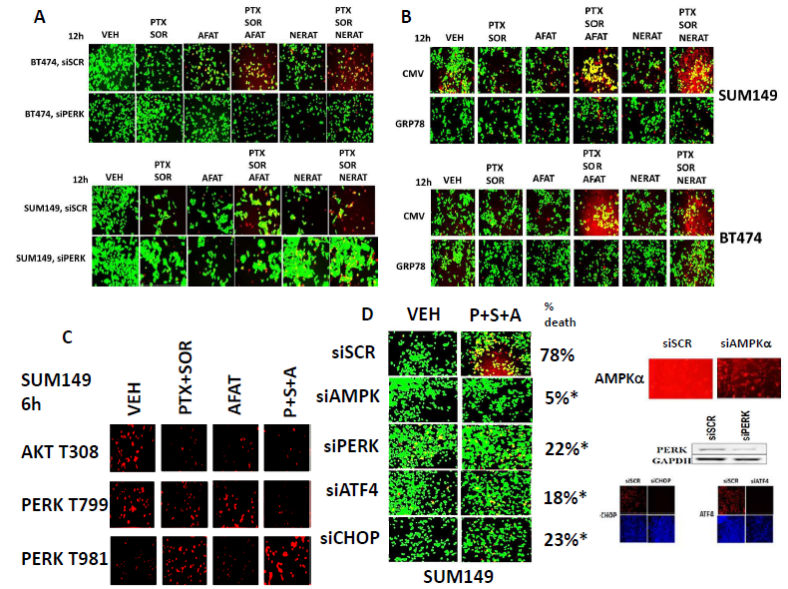
Killing by [pemetrexed + sorafenib + afatinib] requires ER stress signaling as well as inactivation of AKT and PERK dephosphorylation **A.** BT474 and SUM149 cells were transfected with a scrambled siRNA or with an siRNA molecule to knock down the expression of PERK. Twenty four h after transfection cells were treated with vehicle control, [pemetrexed (0.5 μM) and sorafenib (2 μM)], Afatinib (0.5 μM), Neratinib (0.5 μM) or the drugs in combination as indicated. Twelve h after treatment cells were treated with live / dead reagent and cells examined using a Hermes WiScan microscope where red/yellow cells = dead; green cells = alive. **B.** BT474 and SUM149 cells were transfected with an empty vector plasmid (CMV) or with a plasmid to express GRP78. Twenty four h after transfection cells were treated with vehicle control, [pemetrexed (0.5 μM) and sorafenib (2 μM)], Afatinib (0.5 μM), Neratinib (0.5 μM) or the drugs in combination as indicated. Twelve h after treatment cells were treated with live / dead reagent and cells examined using a Hermes WiScan microscope where red/yellow cells = dead; green cells = alive. **C.** SUM149 cells were treated for 6h with vehicle control, [pemetrexed (0.5 μM) and sorafenib (2 μM)], Afatinib (0.5 μM) or the drugs in combination as indicated. Cells were fixed and permeabilized *in situ* and immuno-fluorescence was performed to determine the total phosphorylation level at 10X magnification of PERK T981; PERK T799; and AKT T308. **D.** SUM149 cells were transfected with a scrambled siRNA or with siRNA molecules to knock down the expression of AMPKα1/α2, PERK, ATF4 or CHOP. Twenty four h after transfection cells were treated with vehicle control, [pemetrexed (0.5 μM) and sorafenib (2 μM)], Afatinib (0.5 μM) or the drugs in combination as indicated. Twelve h after treatment cells were treated with live / dead reagent and cells examined using a Hermes WiScan microscope where red/yellow cells = dead; green cells = alive (+/− SEM) * *p* < 0.05 less than siSCR control value.

We then explored the roles of GRP78 and endoplasmic reticulum signaling in drug combination lethality. Molecular knock down of PERK expression protected tumor cells from ERBB1/2 inhibitor + [pemetrexed + sorafenib] lethality (Figure 8A). Over-expression of the PERK inhibitory chaperone GRP78 suppressed drug-induced eIF2α phosphorylation and blocked tumor cell killing by the drug combinations (Figure 8B, data not shown). The data in Figure 8A correlated with the drug combination reducing AKT T308 phosphorylation and reducing the phosphorylation of PERK at T799, a site of AKT-induced inhibitory phosphorylation on PERK, and increasing PERK T981 phosphorylation, a site of stimulatory auto-phosphorylation, which collectively will cause a hyper-activation of PERK (Figure 8C). Knock down of PERK, ATF4 or CHOP expression, both upstream and downstream effectors of eIF2α, also reduced tumor cell death (Figure 8D). Knock down of the AMP dependent protein kinase alpha subunit suppressed drug combination lethality in agreement with prior data showing pemetrexed stimulates ZMP production [1, 2].

**Figure 9:**
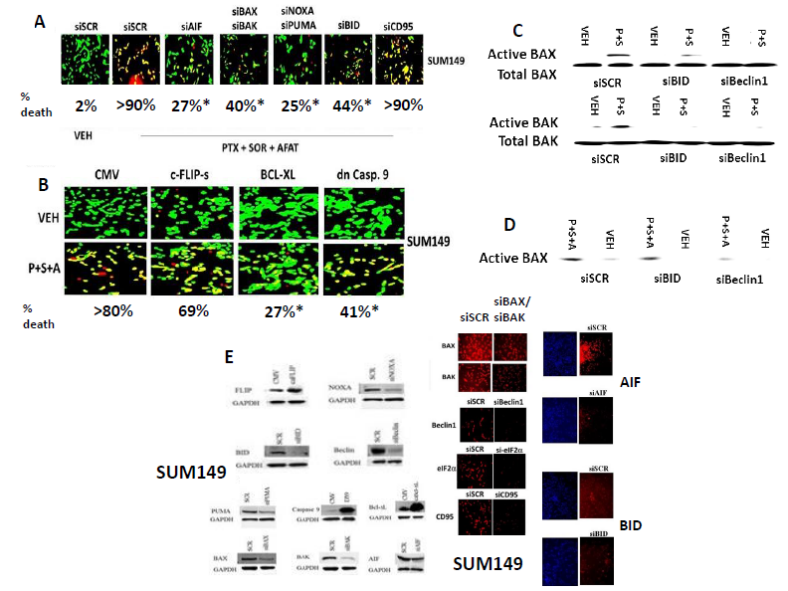
Knock down of [BAX + BAK], [NOXA + PUMA], AIF or over-expression of BCL-XL protects tumor cells from [pemetrexed + sorafenib + afatinib] toxicity **A.** SUM149 cells were transfected with a scrambled siRNA molecule or with the siRNA molecules shown in the Figure. Twenty four h after transfection cells were treated with vehicle control, [pemetrexed (0.5 μM) and sorafenib (2 μM) and afatinib (0.5 μM) in combination as indicated. Twelve h after treatment cells were treated with live / dead reagent and cells examined using a Hermes WiScan microscope where red/yellow cells = dead; green cells = alive (+/− SEM). * *p* < 0.05 less than siSCR value. **B.** SUM149 cells were transfected with an empty vector plasmid or with the protein expression plasmids shown in the Figure. Twenty four h after transfection cells were treated with vehicle control, [pemetrexed (0.5 μM) and sorafenib (2 μM) and afatinib (0.5 μM) in combination as indicated. Twelve h after treatment cells were treated with live / dead reagent and cells examined using a Hermes WiScan microscope where red/yellow cells = dead; green cells = alive. alive (+/− SEM). * *p* < 0.05 less than siSCR value. **C.** and **D.** SUM149 cells were transfected with a scrambled siRNA molecule or with the siRNA molecules shown in the Figure to knock down Beclin1 or BID. Twenty four h after transfection cells were treated with vehicle control, [pemetrexed (0.5 μM) and sorafenib (2 μM) and afatinib (0.5 μM) in combination as indicated. Cells were isolated and broken down using a CHAPS based lysis buffer with vigorous trituation, followed by lysate clarification via centrifugation (5 min × 14,000 g) Equal protein mass portions of lysates were immuno-precipitated using antibodies that recognize epitopes in BAX and in BAK only open to detection when these toxic BH3 domain proteins are active. Immuno-precipitates of active BAX and of active BAK are run on SDS PAGE (14%) and electrophoresed proteins transferred to 0.22 μm thick nitro-cellulose membranes for immuno-blotting against the total expression of BAX and of BAK under each condition. In parallel, a small portion of cell lysate is run alongside the immuno-precipitate as a control for total BAX/BAK expression. **E.** SUM149 cells were transfected with the indicated siRNA molecules and 24h after transfection the expression of the indicated proteins was determined by immuno-fluorescence. SUM149 cells were transfected with plasmids to make the indicated proteins 24h after transfection the expression of the indicated proteins was determined by immuno-fluorescence.

Combined knock down of BAX+BAK or NOXA+PUMA or of AIF alone, or over-expression of BCL-XL partially protected cells from [pemetrexed + sorafenib + afatinib] -induced killing (Figures 9A and 9B). Knock down of CD95 or over-expression of c-FLIP-s did not reduce drug-induced cell killing which is in general congruence with our prior findings that CD95 null HuH7 hepatoma cells were effectively killed by the drug combination [6]. AIF appeared to play a greater role in mediating death signals down-stream of mitochondria than did caspase 9. Also notable was that BID activation down-stream of autolysosomes appeared to be playing a greater role in cell killing than caspase 8 signaling, suggesting cathepsin and calpain proteases rather than caspase 8 were causing BID cleavage. Knock-down of Beclin1 or of BID suppressed the [pemetrexed + sorafenib] -induced activations of BAX and of BAK (Figure 9C). However, knock down of BID did not block BAX activation caused by [pemetrexed + sorafenib + afatinib], despite knock down of Beclin1 still reducing BAX activation (Figure 9D). These findings argue that [pemetrexed + sorafenib] and [pemetrexed + sorafenib + afatinib] -induced stimulation of autophagosome / autolysosome formation plays a central role, upstream of mitochondria and toxic BH3 domain proteins, by which these drug combinations drive forwards tumor cell killing. Confirmatory knock down assessments for the proteins are presented in Figure 9E.

**Figure 10:**
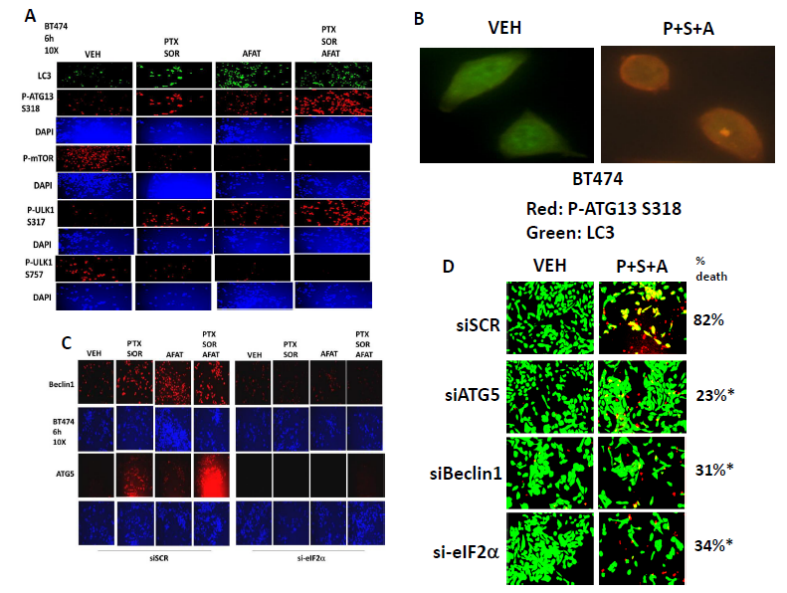
Pemetrexed + Sorafenib + Afatinib promote autophagy through ER stress -induced expression of Beclin1 and LC3, and by mTOR inactivation and AMPK activation leading to ATG13 phosphorylation **A.** BT474 cells were treated with vehicle control, [pemetrexed (0.5 μM) and sorafenib (2 μM)], afatinib (0.5 μM), or the drugs in combination as indicated. Six h after drug treatment cells were fixed and permeabilized *in situ* and immuno-fluorescence was performed to determine the levels of: P-mTOR S2448; P-ULK-1 S757; P-ULK-1 S317; P-ATG13 S318. **B.** BT474 cells were treated with vehicle control or with [pemetrexed (0.5 μM) + sorafenib (2 μM) + afatinib (0.5 μM)] in combination as indicated. Six h after drug treatment cells were fixed and permeabilized *in situ* and immuno-fluorescence was performed at 60X magnification to determine the co-localization of phosphorylated ATG13 (red fluorescent stain) and LC3 (green fluorescent stain). **C.** BT474 cells were transfected with a scrambled siRNA or an siRNA to knock down expression of eIF2α. Twenty four h after transfection cells were treated with vehicle control, [pemetrexed (0.5 μM) and sorafenib (2 μM)], afatinib (0.5 μM), or the drugs in combination as indicated. Six h after drug treatment cells were fixed and permeabilized *in situ* and immuno-fluorescence was performed to determine the levels of: Beclin1 and ATG5. **D.** BT474 cells were transfected with a scrambled siRNA or with siRNA molecules to knock down expression of Beclin1, ATG5 or eIF2α. Twenty four h after transfection cells were treated with vehicle control, [pemetrexed (0.5 μM) and sorafenib (2 μM)], afatinib (0.5 μM), or the drugs in combination as indicated. Cell viability was determined 12h after drug exposure (+/− SEM) * *p* < 0.05 less than corresponding value in siSCR treated.

As the drug combinations were increasing vesicle / autophagosome levels that were an essential component in tumor cell killing, we next investigated the upstream signaling pathways that regulate autophagy. Treatment of cells with [pemetrexed + sorafenib] or [pemetrexed + sorafenib + afatinib] reduced mTOR S2448 phosphorylation and decreased phosphorylation of ULK-1 S757, which is the site in ULK-1 phosphorylated by mTOR to keep the ULK-1 kinase inactive (Figure 10A). The drug combination treatments with pemetrexed also increased ULK-1 S317 phosphorylation, which is one known site of AMP-dependent protein kinase (AMPK) phosphorylation, and is a site whose phosphorylation acts to that stimulate ULK-1 activity. The observed changes in both ULK-1 phosphorylation sites correlated with increased ATG13 S318 phosphorylation (Figure 10A). At 60X magnification co-localization of LC3 (ATG8) and phospho-S318 ATG13 was observed after [pemetrexed + sorafenib] or [pemetrexed + sorafenib + afatinib] treatment (Figure 10B). Treatment of cells with [pemetrexed + sorafenib] or [pemetrexed + sorafenib + afatinib] increased total Beclin1 and ATG5 expression in an eIF2α -dependent manner (Figure 10C). Knock down of Beclin1 or ATG5 or eIF2α reduced the lethality of [pemetrexed + sorafenib + afatinib] (Figure 10D). Thus the drug combinations: [pemetrexed + sorafenib] +/− ERBB1/2/4 inhibitor facilitate a toxic form of autophagy both by increasing autophagy regulatory Beclin1 / ATG5 protein expression through endoplasmic reticulum stress signaling and by also reducing GRP78 and HSP27 chaperone functions thereby differentially altering protein phosphorylation of mTOR / ULK-1 / ATG13 collectively, to facilitate toxic autophagosome / autolysosome formation.

**Figure 11:**
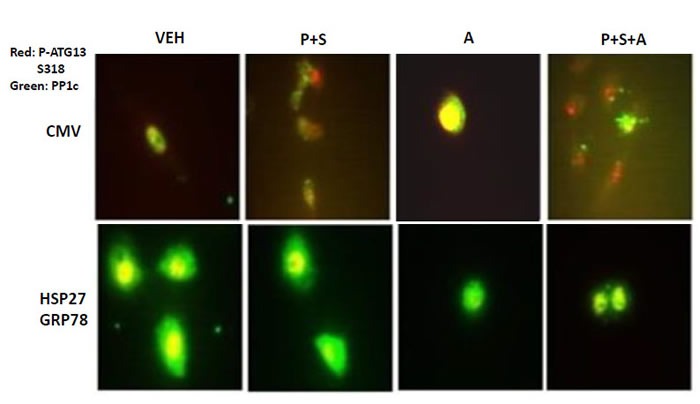
GRP78 and HSP27 regulate the co-localization of PP1c and ATG13 SUM149 cells were transfected with an empty vector plasmid (CMV) or plasmids to express GRP78 and HSP27. Twenty four h after transfection cells were treated for 3h with vehicle control, [pemetrexed (0.5 μM) and sorafenib (2 μM)], afatinib (0.5 μM), or the drugs in combination as indicated. After 3h cells were fixed and permeabilized *in situ* and immuno-fluorescence was performed to determine the co-localization at 60X magnification of P-ATG13 S318 (red) and PP1c (green).

The phosphorylation of proteins is not only dependent upon the actions of protein kinases, but also on those of protein phosphatases [26, 27]. It has been shown that the catalytic subunit of serine / threonine protein phosphatase 1, PP1c, localizes to autophagosomes through its targeting subunit ATG16L1 [28–30]. Six h after treatment of vector control transfected SUM149 cells with [pemetrexed + sorafenib], PP1c and phospho-ATG13 co-localized, as judged by the increase in the yellow fluorescent signal (Figure 11). The co-localization of PP1c with phospho-ATG13 declined in vector control transfected cells treated with [pemetrexed + sorafenib + afatinib]. Over-expression of [GRP78 + HSP27] increased the basal levels of PP1c expression and maintained the co-localization of PP1c with phospho-ATG13 under all treatment conditions. Thus the GRP78 and HSP27 chaperones, by increasing and maintaining PP1c association with ATG13, prevent drug-stimulated autophagic flux towards tumor cell death.

**Figure 12:**
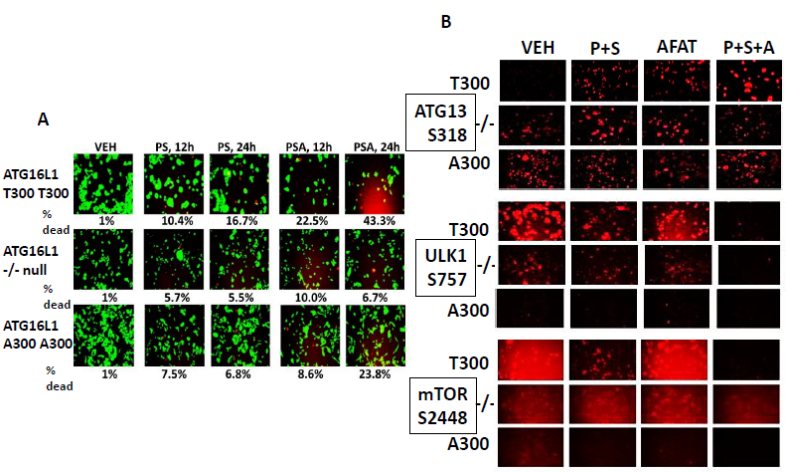
Genetic variants of ATG16L1 alter the lethality of [pemetrexed + sorafenib] +/− afatinib to kill colon cancer tumor cells **A.** HCT116 clones were treated with vehicle control, [pemetrexed (0.5 μM) and sorafenib (2 μM)], afatinib (0.5 μM) or the drugs in combination as indicated. Twelve h after treatment cells were treated with live / dead reagent and cells examined using a Hermes WiScan microscope where red/yellow cells = dead; green cells = alive. **B.** HCT116 clones were treated with vehicle control, [pemetrexed (0.5 μM) and sorafenib (2 μM)], afatinib (0.5 μM) or the drugs in combination as indicated. Twelve h after treatment cells were fixed and permeabilized *in situ* and immuno-fluorescence was performed to determine the total phosphorylation levels at 10X magnification of ATG S318; ULK1 S757; mTOR S2448.

Phosphorylation of ATG16L1 at S139 by casein kinase 2 has been postulated as one mechanism by which PP1c dissociates from the autophagosome, enhancing ATG13 S318 phosphorylation and as a result autophagosome formation [28]. A threonine 300 to alanine 300 variation in ATG16L1, which has a ~50% penetrance in European populations but is much less frequently found in African-American populations (~5%), has been implicated in the development of Crohn's Disease and an inability of bacteria to be phagocytosed by cells and then disposed of by digestion in autophagic vesicles [31, 32]. In cell viability assays, treatment of wild type HCT116 cells that are homozygous for ATG16L1 T300 T300 with [pemetrexed + sorafenib] resulted in high levels of tumor cell killing as we have generally observed in many tumor cell types (Figure 12A). In isogenic ATG16L1 −/− null cells, that are autophagy incompetent, the lethality of [pemetrexed + sorafenib] was, as expected, significantly reduced. In isogenic HCT116 cells that have been genetically modified to homozygously express the ATG16L1 variant A300; [pemetrexed + sorafenib] killed these HCT116 A300 cell variants with the levels of cell killing being significantly lower than observed in HCT116 T300 T300 cells, with values intermediate between those found in ATG16L1 T300 T300 cells and in the −/− null cells (*p* < 0.05).

Based on these findings, we determined in the isogenic HCT116 cells the basal and drug-induced changes in the phosphorylation of ATG13 S318; ULK1 S757; and mTOR S2448. The basal phosphorylation of ATG13 S318 was low in wild type HCT116 cells that express ATG16L1 T300 T300 (Figure 12B). This correlated with high basal phosphorylation of mTOR S2448 and of ULK1 S757. In ATG16L1 null −/− cells, the basal phosphorylation of mTOR and ULK1 was reduced, and the basal phosphorylation of ATG13 S318 increased. In ATG16L1 A300 A300 cells, the basal phosphorylation of mTOR and ULK1 was very low and the phosphorylation of ATG13 high. Treatment of HCT116L1 T300 T300 cells with [pemetrexed + sorafenib] or [pemetrexed + sorafenib + afatinib] reduced ULK1 S757 phosphorylation and mTOR S2448 phosphorylation and increased ATG13 S318 phosphorylation. In contrast, treatment of HCT116 ATG16L1 A300 A300 cells with [pemetrexed + sorafenib] or [pemetrexed + sorafenib + afatinib] only modestly reduced mTOR and ULK1 phosphorylation and had no apparent effect on ATG13 S318 phosphorylation.

One hypothetical way to explain more completely these protein phosphorylation findings would be if the localization of PP1c, *via* ATG16L1, with the autophagosome, changed during drug exposure, as we very likely were observing in the SUM149 breast cancer cells. Under basal conditions in both ATG16L1 T300 T300 and ATG16L1 A300 A300 cells, we discovered that PP1c co-localized with phospho-ATG13 S318, with an apparent greater level of PP1c-ATG13 co-localization in T300 T300 cells compared to the A300 A300 cells as judged by the co-localization signal being a reddish orange-yellow (Figures S1-S3). Upon exposure of ATG16L1 T300 T300 cells to [pemetrexed + sorafenib] the co-localization of PP1c with ATG13 S318 was reduced as judged by the co-localization signal becoming a reddish orange-yellow similar to that observed in vehicle control A300 A300 cells. Exposure of ATG16L1 T300 T300 cells to [pemetrexed + sorafenib + afatinib] abolished the co-localization of PP1c with ATG13 S318, whereas in ATG16L1 A300 A300 cells PP1c still co-localized with ATG13 S318 albeit at a reduced level. Collectively, these data would suggest that the phosphorylation of ATG16L1 Threonine 300 plays a very important role in regulating PP1c localization within the autophagosome complex after [pemetrexed + sorafenib +/− afatinib] exposure. As cells expressing ATG16L1 T300 T300, the variant predominantly found in African-Americans, respond more favorably to [pemetrexed + sorafenib +/− afatinib] than ATG16L1 A300 A300 cells, a phenotype more predominantly found in European-Americans; our findings may have wider societal implications for the delivery of pemetrexed based or other autophagy utilizing cancer therapies.

**Figure 13:**
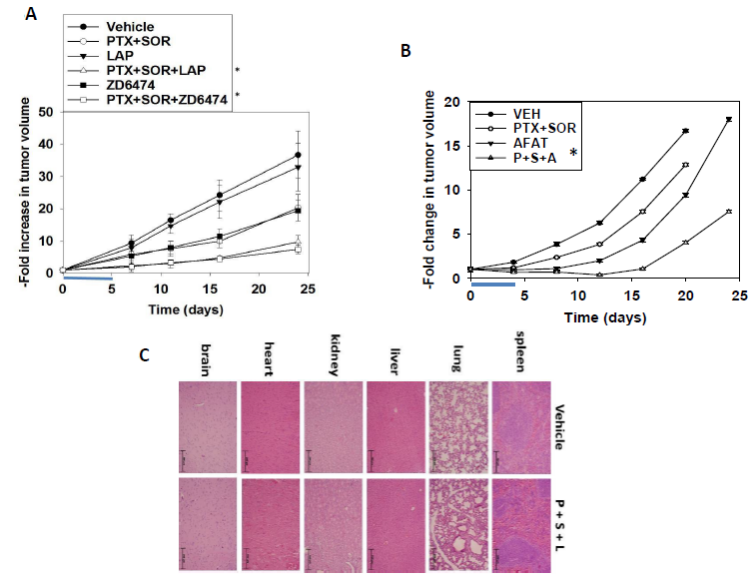
[Pemetrexed + Sorafenib] treatment extends animal survival and is enhanced by inhibition of ERBB1/2 signaling **A.** BT474 cells (1 × 10^7^) were infused into the 4^th^ mammary fat pad of athymic mice and permitted to establish for 7 days. Animals were treated for 5 days with vehicle diluent (cremophore); sorafenib, pemetrexed, lapatinib or ZD6474 (vandetanib), or in the indicated combinations by oral gavage for 5 days. (*n* = 8-10 per group +/− SEM) * *p* < 0.05 lower tumor volume than pemetrexed + sorafenib treatment alone. **B.** H1975 cells (1 × 10^7^) were infused into rear flank of athymic mice and permitted to establish for 7 days. Animals were treated for 5 days with vehicle diluent (cremophore); sorafenib, pemetrexed, afatinib, or in the indicated combinations in the Methods by oral gavage for 5 days. (*n* = 10 per group +/− SEM) * *p* < 0.05 lower tumor volume than pemetrexed + sorafenib treatment alone. **C.** Animals from *panel A*, at the time of sacrifice 19 days after cessation of drug treatment, were dissected and their “normal” tissues obtained. Tissues were fixed, sectioned and H&E stained.

We next determined whether the ERBB1/2/4 inhibitors lapatinib and afatinib or the VEGFR2/ERBB1/4 inhibitor vandetanib could enhance [pemetrexed + sorafenib] toxicity in human breast and human lung cancer models. Using orthotopic BT474 HER2+ human mammary tumors in an athymic mouse, a single 5 day treatment cycle with lapatinib or vandetanib (ZD6474) enhanced the anti-tumor effects of [pemetrexed + sorafenib] (Figure 13A, *p < 0.05). Using H1975 double mutant active ERBB1 T790M L858R expressing non-small cell lung cancer cells growing in athymic mice, a single shorter 4 day treatment cycle with afatinib enhanced the anti-tumor effects of [pemetrexed + sorafenib] (Figure 13B, *p < 0.05). Cell killing was specific to tumor cells, and treatment of animals with [pemetrexed + sorafenib + lapatinib] for 5 days did not alter animal body mass or cause normal tissue damage as judged by H&E staining (Figure 13C, data not shown). In the phase I trial, daily treatment of sorafenib with Q 2 week pemetrexed resulted in cumulative toxicities in some patients who remained on the trial for > 6 months, and the RP2D and schedule for the phase II trial is for pemetrexed Q 2 weeks with sorafenib dosing on days 1-5. Should our proposed three-drug studies eventually move to the clinic it is probable that the dosing of patients with pemetrexed, sorafenib and afatinib, all of which can cause GI dysfunction and reduce blood cell production as well as skin and mouth lesions (afatinib, sorafenib), will again need to be carefully planned so as not to elicit dose-limiting-toxicities within the first cohort / dosing level. Furthermore, because of these issues, and considering the liver metabolism and excretion of the drugs, it is possible that the PK / PD of each agent will be modified by the inclusion of each of the other agents; as we have observed alterations in ABCB1 and ABCG2 expression, altered drug accumulation in normal tissues may also become an issue in the design of any new phase I trial.

**Figure 14:**
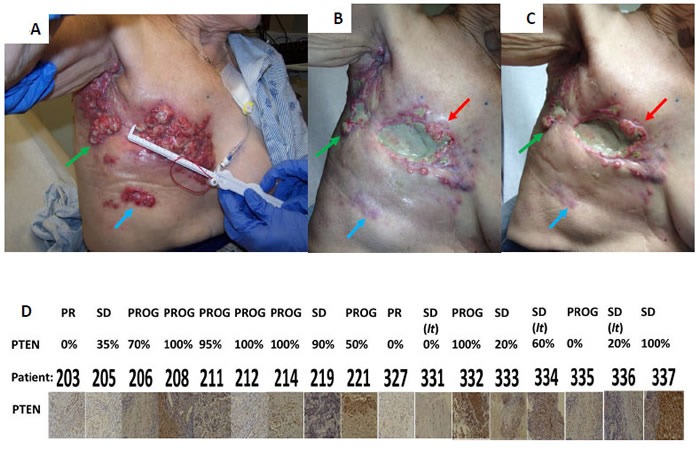
Individual clonal re-growth of mammary carcinoma in a patient treated with [pemetrexed + sorafenib] **A.**-**C.** This triple negative mammary carcinoma patient had received 10 prior therapies before recruitment onto the phase I trial NCT01450384, and was the second patient on the trial at the lowest dose level (200 mg BID sorafenib; 500 mg / m^2^ pemetrexed). Data in Panel A shows the extent of the cutaneous tumor prior to therapy. Data in Panel B shows the response of the tumor four months after the start of therapy. Data in Panel C shows the response of the tumor after 5 months, and with the start of some clonal tumor re-growth. The colored arrows, indicate tumors that responded with either a CR (blue), stable disease (green), or a PR followed by disease progression (red). **D.** Pre-treatment paraffin embedded tumor material from each patient was sectioned, de-paraffinized and subjected to antigen retrieval. All slides had previously been immuno-stained using DAB conversion to detect PTEN protein expression levels [20]. Visible light images of DAB staining for PTEN is presented.

As reported at the 2015 ASCO meeting, treatment of recurrent solid tumor patients with [pemetrexed + sorafenib] resulted in ~60% of all patients experiencing some degree of tumor growth delay (SD, PR, CR), with multiple partial responses and one complete response (NCT01450384) [20]. In our phase I trial we found that even within a large tumor that profoundly responded to treatment, individual portions of the tumor displayed various forms of response against the [pemetrexed + sorafenib] drug combination (Figures 14A-14C). The patient in Figure 14 was treated at the lowest dose level of 500 mg/m^2^ pemetrexed and 200 mg BID sorafenib. The patient in Panel A is a pre-treatment image of a triple negative mammary carcinoma that had previously received 10 prior therapeutic interventions; the image in Panel B is after 4 months of [pemetrexed + sorafenib] therapy; that in Panel C is after 5 months of [pemetrexed + sorafenib] therapy when the patient had to leave the trial due to tumor progression. The tumor labeled with the blue arrow completely resolved after treatment whereas the portion of the tumor labeled with the green arrow displayed a cyto-static effect. The tumor material labeled with the red arrow initially strongly responded to the drug combination, but note that portions of the tumor then displayed re-growth after 5 months. From this data we conclude that any anti-cancer drug combination, even one as multi-mechanistic and as potent as [pemetrexed + sorafenib], which is designed to only kill a highly specific subset of tumor cells i.e. the idealized “personalized medicine approach” that is being touted by the medical community to the public at large, will be unlikely to provide any true long-term / curative benefit to the majority of patients who carry multiple clonal variants of the same tumor at the same time.

As part of NCT01450384, sections of pre-therapy archived tumor blocks from patients were subjected to immuno-histochemistry by a certified pathologist to determine the basal expression levels of the tumor suppressor PTEN. Differences were seen in the expression levels of PTEN between patients, with some tumors exhibiting very high levels; some low levels; and some tumors that were PTEN null (Figure 14D). There are a very large number of studies over the last 25 years which that state loss of PTEN results in resistance to many diverse forms of chemotherapy and radiotherapy; the clinical observation that a reduction or loss of PTEN expression significantly correlates with *enhanced therapeutic effects* of any drug combination has not been reported. The ordinal regression statistical results from the trial indicate a significant association between no or low expression levels of PTEN and patient response (*p* = 0.013) [20].

As part of the studies in Figure 13B, we also generated by *in vivo* high dose afatinib (50 mg/kg BID) for 4 days treatment afatinib resistant H1975 cells (5 tumors / clones of control; 5 tumors / clones of afatinib resistant) [34, 35]. Afatinib caused H1975 flank tumors to completely disappear for approximately one week and then the tumors slowly began to re-grow. We then performed multiplex assays on portions of tumor tissue from tumors in Figure 13B as well as the afatinib resistant tumors taken at the time of mouse sacrifice due to tumor volume after ~30 days. Afatinib resistant H1975 tumors contained higher expression levels of the human cytokines CXCL-1, IL-6 and IL-8 and expressed higher basal levels of the IL-8 receptor (Figure S4A). Knock down of the IL-8 receptor enhanced [pemetrexed + sorafenib] lethality in afatinib resistant H1975 cells. Afatinib resistant H1975 tumors contained higher levels signaling within their PI3K and NFκB pathways compared to control tumors (Figures S4A-S4D). Based on the multiplex data showing increased PI3K pathway activity in afatinib resistant H1975 clones and in [pemetrexed + sorafenib + afatinib] resistant clones we next determined whether inhibition of PI3K signaling enhanced [pemetrexed + sorafenib] lethality and whether any changes in PTEN expression and its Serine 380 regulatory phosphorylation could be found in our *in vivo* generated afatinib resistant H1975 clones. Inhibition of PI3K signaling using the phase III PI3Kα/β/γ/δ inhibitor drug copanlisib enhanced [pemetrexed + sorafenib] toxicity in afatinib-resistant H1975 clones to a greater extent than it did in control H1975 clones (Figure S4E, *p* < 0.05).

Expression of PTEN was considerably reduced in afatinib resistant H1975 tumor clones compared to control clones that correlated with elevated AKT T308 phosphorylation (Figure S5A). Afatinib resistant H1975 clones also exhibited lower levels of PTEN S380 phosphorylation compared to control clones. Based on image density analyses the ratio of PTEN S380 phosphorylation to total PTEN expression was 0.31 in control clones which was significantly lower than the ratio found in afatinib resistant cells which was 0.52 (*p* < 0.05). This finding shows afatinib resistant H1975 clones, by virtue of expressing less PTEN and having a greater proportion of this PTEN phosphorylated at Serine 380 and thus inactive, will permit more active signaling from PI3K through PTEN and downstream into AKT / mTOR than will control clones.

Afatinib resistant clones had higher basal expression of Beclin1, lower expression of PP2Ac and CHOP, and higher basal phosphorylation of ULK-1 S317, ULK-1 S757 and ATG13 S318, which correlated with elevated autophagosome levels (Figures S5B and S5C). Treatment of control and afatinib resistant clones with [pemetrexed + sorafenib] or [pemetrexed + sorafenib + afatinib] reduced mTOR S2448 phosphorylation and decreased phosphorylation of ULK-1 S757, which is the site in ULK-1 phosphorylated by mTOR to keep the kinase inactive (Figure S5D). The observed changes in both ULK-1 phosphorylation sites correlated with increased ATG13 S318 phosphorylation (Figure S5E). Thus the patient tumors most responsive to [pemetrexed + sorafenib] expressed lower PTEN as did afatinib-resistant H1975 tumors; and afatinib synergized with [pemetrexed + sorafenib] to kill *in vitro* and *in vivo*. Previously we noted that tumor cell types that displayed high levels of cell killing after [pemetrexed + sorafenib] exposure, such as MCF7F, H460 and HuH7 tended to exhibit significantly elevated basal levels of AKT, p70 S6K and/or mTOR phosphorylation [6]. Hence we also postulate the possibility that just as a mutated active ERBB1-addicted tumor cell may be attempting to evolve away from afatinib sensitivity by reducing its PTEN levels, it could encounter [pemetrexed + sorafenib] therapy which preferentially kills those cells expressing low PTEN.

Finally, because of these findings we performed additional assays, knocking down PTEN expression in tumor cells and determining whether this altered the killing response to [pemetrexed + sorafenib] and [pemetrexed + sorafenib + afatinib]. Using H1975 lung tumor cells that express a wild type PTEN, knock down of PTEN expression enhanced the lethality of [pemetrexed + sorafenib] but not of [pemetrexed + sorafenib + afatinib] where killing was observed as being > 50% of the cell regardless of the cellular PTEN status. (Figure S6A) [40, 44]. Expression of wild type PTEN in the PTEN null glioblastoma cell line GBM14 reduced the efficacy of [pemetrexed + sorafenib] or [pemetrexed + sorafenib + afatinib] but increased the killing power of afatinib as a single agent (Figure S6B).

The precise reasons why paradoxically both inhibition of PI3K *and* loss of PTEN can both enhance [pemetrexed + sorafenib] lethality will require many additional studies. However, one possible explanation for this effect could be suggested by some additional data hewed from the afatinib resistant H1975 clones. In the afatinib resistant clones, where PTEN levels were low, we noted that expression of the negative transcriptional regulator of the PTEN gene, NEDD4, was much higher than in control clones, and that the expression of another NEDD4 target and PTEN-associated protein, PTPN13 (FAP-1), was also low (Figure S5F) [36]. PTPN13 is a protein tyrosine phosphatase that is localized in the plasma membrane environment by its PDZ domains to PTEN, where PTPN13 was originally described as the tyrosine phosphatase that dephosphorylates and inactivates the death receptor CD95 but subsequently also the proteins PKN2, PI3K p85β, IκBα and Rho-GAP, and reduced PTPN13 expression in afatinib resistant H1975 cells correlated with elevated tyrosine phosphorylation of ERBB3 and SRC Y416. *Nota bene*: as previously shown neither knock down of CD95 nor expression of the caspase 8 inhibitor c-FLIP-s reduced the lethality of [pemetrexed + sorafenib + afatinib] (Figures 9A and 9B). Thus by reducing PTEN levels the plasma membrane localization of other phosphatases is also reduced leading to greater levels of tyrosine phosphorylation -dependent signaling, particularly through the PI3K pathway.

From multiplex assays on tumor material in Figure 13B we discovered that [pemetrexed + sorafenib + afatinib] exposed tumors had activated their PI3K and NFκB pathways. The drug flavopiridol (Alvocidib) is recognized as a CDK7/9 inhibitor but is also a potent inhibitor of IKKα/β [37–40]. Copanlisib is a class I selective PI3K p110α/β/γ/δ inhibitor. H1975 tumor clones from animals previously treated with [pemetrexed + sorafenib + afatinib] exhibited greater sensitivity to both flavopiridol and copanlisib as single agents than did control clones (Figure S6C). In combination, using flavopiridol at two orders lower than its patient plasma C_max_ and using copanlisib at one order lower than its reported C_max_, we noted profound levels of preferential [pemetrexed + sorafenib + afatinib] exposed cell killing with the drug combination but not with the individual drugs. In agreement with this data, expression of dominant negative IκB S32A S36A combined with simultaneous knock down of PI3K p110α/β synergized to kill tumor clones previously exposed to [pemetrexed + sorafenib + afatinib] (Figure S6D). Thus [flavopiridol + copanlisib] subverts secondary drug resistance mechanisms.

In conclusion, in this manuscript we have demonstrated that [pemetrexed + sorafenib] and [pemetrexed + sorafenib + afatinib] resistance mechanisms can be overcome in a rational unbiased fashion by use of multiplex antibody micro-array assays and pre-existing signaling pathway knowledge and analyses. The schematic in Figure S7 attempts to describe the multiple overlapping mechanisms by which [pemetrexed + sorafenib +/− afatinib] interact to kill tumor cells. Sorafenib inhibits the activities of multiple serine / threonine / tyrosine kinases as well as the chaperones GRP78, HSP90 and HSP70 (and other chaperones, e.g. HSP60, data not shown). Loss of GRP78 / HSP90 / HSP70 chaperone functions reduces gross protein translation through elevated ER stress eIF2α signaling leading to lower expression of growth factor receptors such as ERBB2 and mitochondrial protective proteins such as MCL-1, but also increases expression of the autophagosome regulatory proteins Beclin1, ATG5 and LC3. Sorafenib, likely through HSP70 dysregulation and p38 MAPK inhibition, causes inactivation of the chaperone HSP27, which together with loss of GRP78, results in a destabilized PI3K signaling pathway, with lower levels of AKT and mTOR activity. Reduced AKT signaling de-represses the ER stress kinase PERK further facilitating the ER stress response caused by loss of GRP78 function. Reduced mTOR activity results in the dephosphorylation of ULK-1 at serine 757, and pemetrexed treatment through AMPK signaling increases ULK-1 serine 317 phosphorylation which collectively lead to a strong activation of ULK-1 kinase activity with the phosphorylation of ATG13, the gate-keeper protein for autophagosome formation, being strongly elevated. The phosphorylation of ATG13 is further enhanced due to reduced expression and co-localization of the serine / threonine protein phosphatase 1 with ATG16L1. Increased autophagosome / autolysosome flux leads to the release of cathepsins and calpains into the cytosol where they catalyze the cleavage of the toxic BH3 domain protein BID that occurs concomitantly with the ER stress -induced decline in MCL-1 and BCL-XL expression; and, loss of HSP90 and HSP70 function also enables the reduction in expression of these tumor cell protective proteins. The alterations in BID / MCL-1 / BCL-XL function result in the activation of BAX and BAK, and the release of apoptosis inducing factor into the cytosol. Additionally, because the function of HSP70 has been suppressed, AIF more readily translocates from the cytosol to the nucleus where it executes the tumor cell.

The addition of an ERBB1/2/4 inhibitor such as afatinib to [pemetrexed + sorafenib] enhanced the already present activated mechanisms outlined in the last paragraph, including enhanced down-regulation of chaperone expression resulting in enhanced ER stress signaling and also caused a more rapid inactivation of mTOR resulting in greater autophagosome formation. With regard to “toxic autophagy” in our pemetrexed studies, ourselves and others have found that estrogen-dependent MCF7 cells, and many primary diagnosed breast cancers, have a haplotype insufficiency in Beclin1 and thus are less effective at inducing autophagy than non-transformed mammary epithelial cells, although the clinical data from our phase I trial did not show low Beclin1 levels in any heavily pre-treated patient [41]. Hence this would suggest that loss of autophagy in the early development of breast cancer facilitates tumor formation. We previously discovered that fulvestrant resistant MCF7 cells expressed higher basal levels of Beclin1 and ATG5-ATG12, compared to parental MCF7 cells arguing that this cell line in part maintains its viability in the face of estrogen deprivation by inducing a protective form of autophagy, yet this cell line also has much higher basal AKT / mTOR activity compared to control MCF7 cells which on paper would be expected to suppress the induction of autophagy, or autophagic flux, in the fulvestrant resistant cells. These findings also agree with other data showing that chloroquine, which inhibits autophagosome - lysosome fusion, can facilitate the anti-tumor effects of the anti-estrogen tamoxifen [42]. Previously we noted that the fulvestrant resistant MCF7 cells were more susceptible to [pemetrexed + sorafenib] killing which was dependent on the induction of a toxic form of autophagy [6, 43]. Thus collectively, we believe these findings further support the concept that it is the *differential* of the decline in AKT / mTOR signaling per se in real terms which plays the biggest role in the lethal autophagic processes being induced by the pemetrexed based drug combinations, rather than just the drugs simply “suppressing” pathway activities.

Additionally, however, by blocking the compensatory activation of ERBB1 caused by the two-drug [pemetrexed + sorafenib] treatment, afatinib also acts to suppress the compensatory induction of anti-apoptotic SOD2 and the DNA repair enzyme XRCC1. Because of afatinib, [pemetrexed + sorafenib] does not increase SOD2 expression that in turn cannot act to suppress autophagosome formation and the generation of high levels of reactive oxygen species caused by mitochondrial dysfunction. Because XRCC1 induction is blocked by afatinib, DNA damage caused by pemetrexed persists, as judged by prolonged ATM activity, which can have multiple outcomes including: further NFκB activation; tumor cell death; genomic instability or senescence.

As [pemetrexed + sorafenib] has safely passed through phase I evaluation and into phase II specifically for triple negative mammary carcinoma (NCT02624700), the present findings strongly argue that a new phase I trial, in all solid tumor patients, combining [pemetrexed + sorafenib + afatinib] should be contemplated, resources permitting.

## MATERIALS AND METHODS

### Materials

Pemetrexed was purchased from LC Laboratories (Woburn, MA). FTY720 was purchased from Cayman Chemical Inc., (Ann Arbor MI). Sorafenib tosylate and all ERBB receptor and kinase inhibitors were purchased from Selleckchem (Houston, TX). Trypsin-EDTA, DMEM, RPMI, penicillin-streptomycin were purchased from GIBCOBRL (GIBCOBRL Life Technologies, Grand Island, NY). Cells were purchased from the ATCC and were not further validated beyond that claimed by ATCC. Cells were re-purchased every ~6 months. The plasmid to express GRP78/BiP/HSPA5 was kindly provided to the Dent laboratory by Dr. A.S. Lee (University of Southern California, Los Angeles, CA); all other plasmids were purchased from Addgene. Commercially available validated short hairpin RNA molecules to knock down RNA / protein levels were from Qiagen (Valencia, CA) or were supplied by collaborators. Rabbit antiserum for mouse PERK phosphorylated at T799 was developed as described [44]. Reagents and performance of experimental procedures were described in refs: [12/13/17/18/21–24/33/40].

### Methods

#### Culture and *in vitro* exposure of cells to drugs

All cell lines were cultured at 37°C (5% (v/v CO_2_) *in vitro* using RPMI supplemented with dialyzed 5% (v/v) fetal calf serum and 10% (v/v) Non-essential amino acids. Cells growing in “complete” fetal calf serum that contains thymidine were gradually weaned into dialyzed serum lacking thymidine over 2 weeks and were then used for experimental analyses for the following 3 weeks before discarding. Cells were re-isolated in thymidine-less media as required. For short term cell killing assays, immunoblotting studies, cells were plated at a density of 3 × 10^3^ per cm^2^ (~2 × 10^5^ cells per well of a 12 well plate) and 48h after plating treated with various drugs, as indicated. *In vitro* pemetrexed, sorafenib and other drug treatments were generally from a 100 mM stock solution of each drug and the maximal concentration of Vehicle carrier (VEH; DMSO) in media was 0.02% (v/v). Cells were not cultured in reduced serum media during any study in this manuscript.

### Transfection of cells with siRNA or with plasmids

#### For plasmids

Cells were plated and 24h after plating, transfected. Plasmids expressing a specific mRNA (or siRNA) or appropriate vector control plasmid DNA was diluted in 50μl serum-free and antibiotic-free medium (1 portion for each sample). Concurrently, 2μl Lipofectamine 2000 (Invitrogen), was diluted into 50μl of serum-free and antibiotic-free medium (1 portion for each sample). Diluted DNA was added to the diluted Lipofectamine 2000 for each sample and incubated at room temperature for 30 min. This mixture was added to each well / dish of cells containing 200μl serum-free and antibiotic-free medium for a total volume of 300 μl, and the cells were incubated for 4 h at 37¼C. An equal volume of 2x medium was then added to each well. Cells were incubated for 24h, then treated with drugs.

#### Transfection for siRNA

Cells from a fresh culture growing in log phase as described above, and 24h after plating transfected. Prior to transfection, the medium was aspirated and serum-free medium was added to each plate. For transfection, 10 nM of the annealed siRNA, the positive sense control doubled stranded siRNA targeting GAPDH or the negative control (a “scrambled” sequence with no significant homology to any known gene sequences from mouse, rat or human cell lines) were used. Ten nM siRNA (scrambled or experimental) was diluted in serum-free media. Four μl Hiperfect (Qiagen) was added to this mixture and the solution was mixed by pipetting up and down several times. This solution was incubated at room temp for 10 min, then added drop-wise to each dish. The medium in each dish was swirled gently to mix, then incubated at 37°C for 2h. Serum-containing medium was added to each plate, and cells were incubated at 37°C for 24h before then treated with drugs (0-24h). Additional immuno-fluorescence / live-dead analyses were performed at the indicated time points.

### Animal studies (breast cancer)

Athymic nude mice (~20 g) were injected with 1 × 10^7^ BT474 cells into their fourth mammary fat pad (8-10 animals per treatment group; 6 groups; a total of 54 mice +/− SEM). Alternatively, BALB/c immune competent mice (~20 g) were injected with 2 × 10^4^ 4T1 cells in their fourth mammary fat pad. Tumors were permitted to form in both animal models for 7 days with tumors at that time exhibiting a mean volume of ~25 mm^3^. For BT474, athymic mice were treated by oral gavage once every day for four days as indicated in the Figure and Figure Legend with vehicle (Cremophore); with pemetrexed (50 mg/kg) only on day 1; with sorafenib tosylate (25 mg/kg) on days 1-4; with lapatinib (50 mg/kg) on days 1-4; with vandetanib (25 mg/kg) on days 1-4. For studies with 4T1 cells, BALB/c mice were treated by oral gavage once every day for five days as indicated in the Figure and Figure Legend with vehicle (Cremophore) or with pemetrexed (50 mg/kg) only on day 1; with sorafenib tosylate (25 mg/kg) on days 1-5. After cessation of drug treatment tumors are again calipered and tumor volume was assessed up to 35 days later.

### Animal studies (lung cancer)

Athymic nude mice (~20 g) were injected with 1 × 10^7^ H1975 cells into their rear flank (10 animals per treatment group; 4 groups; a total of 40 mice +/− SEM). Tumors were permitted to form for 7 days with tumors at that time exhibiting a mean volume of 25-50 mm^3^. Athymic mice were treated by oral gavage once every day QD for four days as indicated in the Figure and Figure Legend with vehicle (Cremophore); with pemetrexed (50 mg/kg) only on day 1; with sorafenib tosylate (25 mg/kg) on days 1-5; with afatinib (10 mg/kg) on days 1-5. After cessation of drug treatment tumors are again calipered and tumor volume was assessed up to 20 days later.

For studies to generate afatinib resistant H1975 cells, pre-existing tumors as above were treated with afatinib (50 mg/kg) BID for 4 days. This reduced tumor volume of all clones to 0 for approximately 7 days after which tumors began to slowly re-grow. Recurrent tumors were isolated on Day 25, portions were snap-frozen or were digested to release individual tumor cells, and cells from each tumor clone maintained separately. Of significant note for clonal characterization, the isolated afatinib treated tumor cells were only growth inhibited by afatinib *in vitro* with daily supplementation at concentrations > > 2 μM, and as such these cells were routinely passaged in a pulsatile fashion between experiments in growth media containing only 1 μM afatinib to maintain the phenotype but not to promote further selective pressure.

### Detection of cell viability, protein expression and protein phosphorylation by immuno-fluorescence using a Hermes WiScan machine

http://www.idea-bio.com/, Cells (4 × 10^3^) are plated into each well of a 96 well plate, and cells permitted to attach and grow for the next 18h. Based on the experiment, after 18h, cells are then either genetically manipulated, or are treated with drugs. For genetic manipulation, cells are transfected with plasmids or siRNA molecules and incubated for an additional 24h. Cells are treated with vehicle control or with drugs at the indicated final concentrations, alone or in combination. Cells are then isolated for processing at various times following drug exposure. The 96 well plate is centrifuged / cyto-spun to associate dead cells (for live-dead assays) with the base of each well. For live dead assays, after centrifugation, the media is removed and cells treated with live-dead reagent (Thermo Fisher Scientific, Waltham MA) and after 10 min this is removed and the cells in each well are visualized in the Hermes instrument at 10X magnification. Green cells = viable; yellow/red cells = dying/dead. The numbers of viable and dead cells were counted manually from three images taken from each well combined with data from another two wells of separately treated cells (i.e. the data is the mean cell dead from 9 data points from three separate exposures). For immuno-fluorescence studies, after centrifugation, the media is removed and cells are fixed in place and permeabilized using ice cold PBS containing 0.4% paraformaldehyde and 0.5% Triton X-100. After 30 min the cells are washed three times with ice cold PBS and cells are pre-blocked with rat serum for 3h. Cells are then incubated with a primary antibody to detect the expression/phosphorylation of a protein (usually at 1:100 dilution from a commercial vendor) overnight at 37°C. Cells are washed three times with PBS followed by application of the secondary antibody containing an associated fluorescent red or green chemical tag. After 3h of incubation the antibody is removed and the cells washed again. The cells are visualized at either 10X or 60X in the Hermes machine for imaging assessments. All immunofluorescent images for each individual protein / phospho-protein are taken using the identical machine settings so that the levels of signal in each image can be directly compared to the level of signal in the cells treated with drugs. Similarly, for presentation, the enhancement of image brightness/contrast using PhotoShop CS6 is simultaneously performed for each individual set of protein/phospho-protein to permit direct comparison of the image intensity between treatments. Antibodies used include: HSP90 (E289) (Cell Signaling); HSP90 (#2928) (Abcam); HSP90 (ab195575) Abcam; HSP90 3G3 (13495) (Abcam); GRP78 (50b12) (31772) (Cell Signaling); GRP78 (ab191023) Abcam; GRP78 (ab103336) Abcam; GRP78 (N-20) (sc-1050) Santa Cruz; HSP27 (G31) (2402P) Cell Signaling); HSP27 [EP1724Y] (ab62339) Abcam; HSP27 (H-77) (sc-9012) Santa Cruz; HSP27 (LS-C31836) Lifespan science Corp. Other antibodies were as used in prior studies by the laboratory. All immunofluorescent images were initially visualized at 75 dpi using an Odyssey infrared imager (Li-Cor, Lincoln, NE), then processed at 9999 dpi using Adobe Photoshop CS6. For presentation, immunoblots were digitally assessed using the provided Odyssey imager software. Images have their color removed and labeled figures generated in Microsoft PowerPoint.

### Multiplex assays for cytokine expression

A Bio-Rad MAGPIX instrument with associated software was purchased from Bio-Rad. The following Bio-Plex assay plates were used in our assays of mouse plasma: PRO Mouse Cyto 23-PLEX (M60009RDPD); PRO TGF-B 3-PLEX (171W4001M); PRO Mouse Cyto Basic FGF set (171G6002M); Mouse Cyto STD GRP II 9-PLEX (171I60001); Mouse Cyto LIF set (171G6003M); Mouse Cyto PDGF-BB set (171G6007M); Mouse Cyto VEGF set (171G6008M). Mouse plasma was assayed according to the instructions provided by Bio-Rad and with Bio-Rad technical assistance.

### Isolation of GST-NH2 terminal HSP90 from bacteria and the measurement of its ATPase activity

*E. coli* BL21 [F-, *ompT*, *hsdS* (rB, mB ), *gal*] were transformed with a plasmid to express a fusion protein of the NH2-terminal portion of HSP90 fused to glutathione-S-transferase (GST) (purchased from Addgene). Bacteria were grown at 24°C with shaking until the A600 reached 0.6. IPTG was added to a final concentration of 0.1 mM and the incubation continued for an additional 6 hours. Bacteria were recovered by centrifugation and stored on ice. The cell pellet was resuspended in 50 μl of ice-cold 1X PBS per ml of culture. The re-suspended cells were mechanically disrupted using a probe sonicator, on ice, in short 5 second bursts. Triton X-100 was added to a final concentration of 1% (v/v) followed by 30 minutes of gentle shaking to aid in solubilization of the fusion protein. Bacterial debris and denatured proteins were removed by centrifugation at 15,000 × g for 20 min at 4°C. The supernatant was removed and immediately mixed with pre-equilibrated Glutathione Sepharose 4B (2 ml of a 50% Sepharose slurry is mixed with 100 ml of clarified bacterial sonicate. The Sepharose slurry is gently rotated in a cold room for 30 min. The slurry mixture is centrifuged (10,000 × g for 10 min) and the supernatant discarded. The Glutathione Sepharose is washed with 10 bead volumes of 1X PBS. The Sepharose slurry is centrifuged (5,000 × g, 5 min), and the supernatant discarded. The Sepharose beads are washed three times. Chaperone ATPase activity using the ATPlite 1step kit (PerkinElmer) was determined using GST-NH2 terminal HSP90 still linked to the Glutathione Sepharose 4B beads. The Sepharose beads are equilibrated in the reaction buffer provided by the manufacturer for 30 min with gentle mixing, and the beads recovered by centrifugation. The beads are then resuspended 1:1 with reaction buffer. To each well of a 96 well plate is added 50 μl of bead slurry and 50 μl of substrate buffer solution containing vehicle control or drug to achieve the desired final concentration. The reactions are started using a multi-channel pipette delivering 50 μl of reconstituted reagent to each well. The plate is placed in foil in an orbital shaker at 37°C for 15 min. The plate is removed; centrifuged to remove floating Sepharose beads; and 100 μl of the supernatant from each well placed into a new well in another 96 well plate. The light emitted from each well / treatment condition is quantified using a Vector 3 plate reader (*n* = 3 of three studies +/− SEM).

### Data analysis

Comparison of the effects of various treatments was performed using one-way analysis of variance and a two tailed Student's *t*-test. Statistical examination of *in vivo* animal survival data utilized log rank statistical analyses between the different treatment groups. Differences with a *p*-value of < 0.05 were considered statistically significant. Experiments shown are the means of multiple individual points from multiple experiments (± SEM).

## SUPPLEMENTARY MATERIALS FIGURES



## Abbreviations

ERK: extracellular regulated kinase
PI3K: phosphatidyl inositol 3 kinase
ca: constitutively active
dn: dominant negative
PTX: pemetrexed
ER: endoplasmic reticulum
mTOR: mammalian target of rapamycin
MAPK: mitogen activated protein kinase
PTEN: phosphatase and tensin homologue on chromosome ten
ROS: reactive oxygen species
CMV: empty vector plasmid
si: small interfering
SCR: scrambled
IP: immunoprecipitation
VEH: vehicle
