# Supplementary material for: [Pemetrexed + Sorafenib] lethality is increased by inhibition of ERBB1/2/3-PI3K-NFκB compensatory survival signaling
Source: Oncotarget. 2016 Mar 22;7(17):23608–32. doi: 10.18632/oncotarget.8281 (PMC5029651; doi:10.18632/oncotarget.8281)
Supplement: Supplementary file 1 [file oncotarget-07-23608-s001.pdf]

[Pemetrexed + Sorafenib] lethality is increased by inhibition of ERBB1/2/3-PI3K-NFκB compensatory survival signaling

Supplementary Material

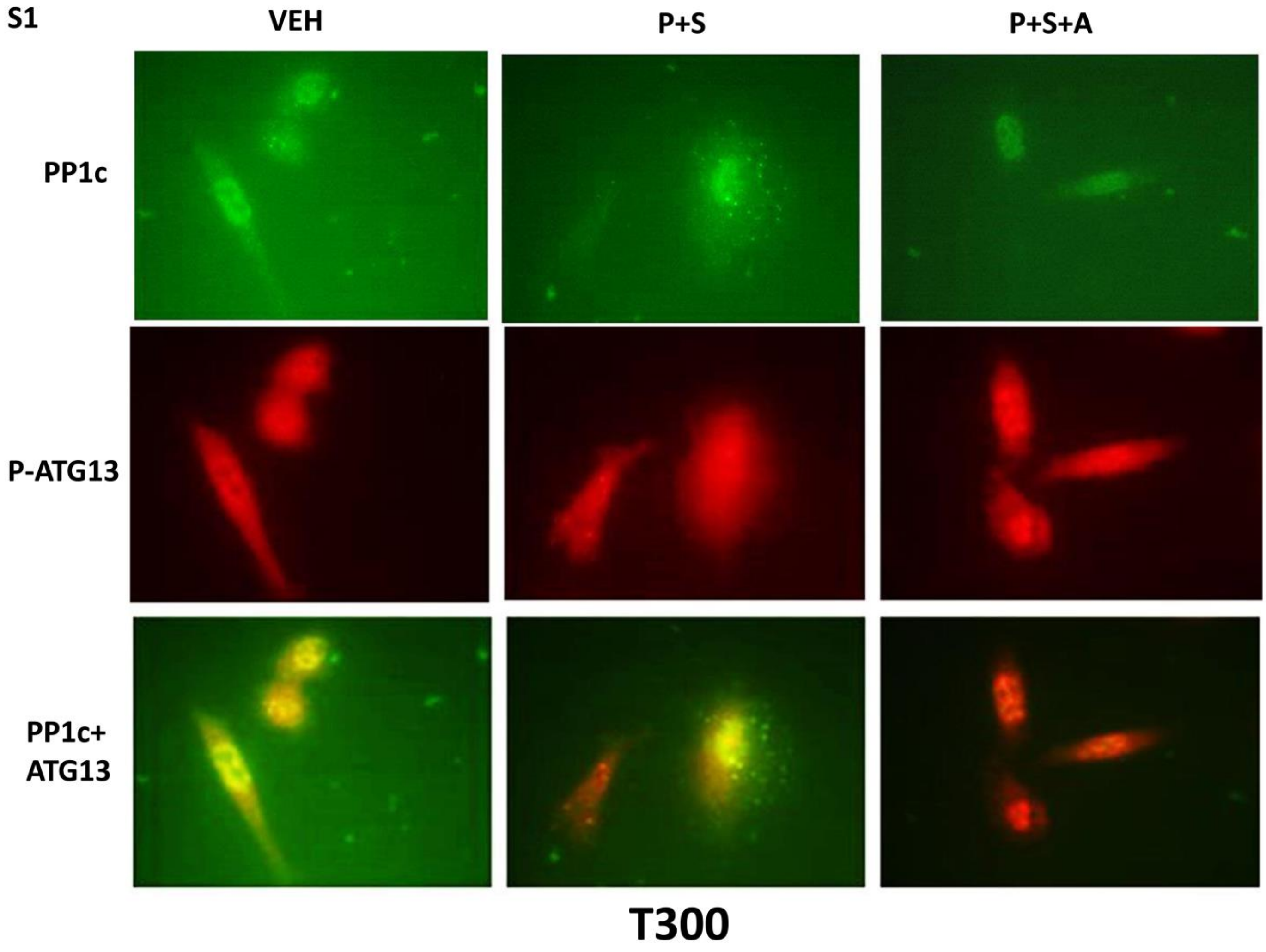

S2

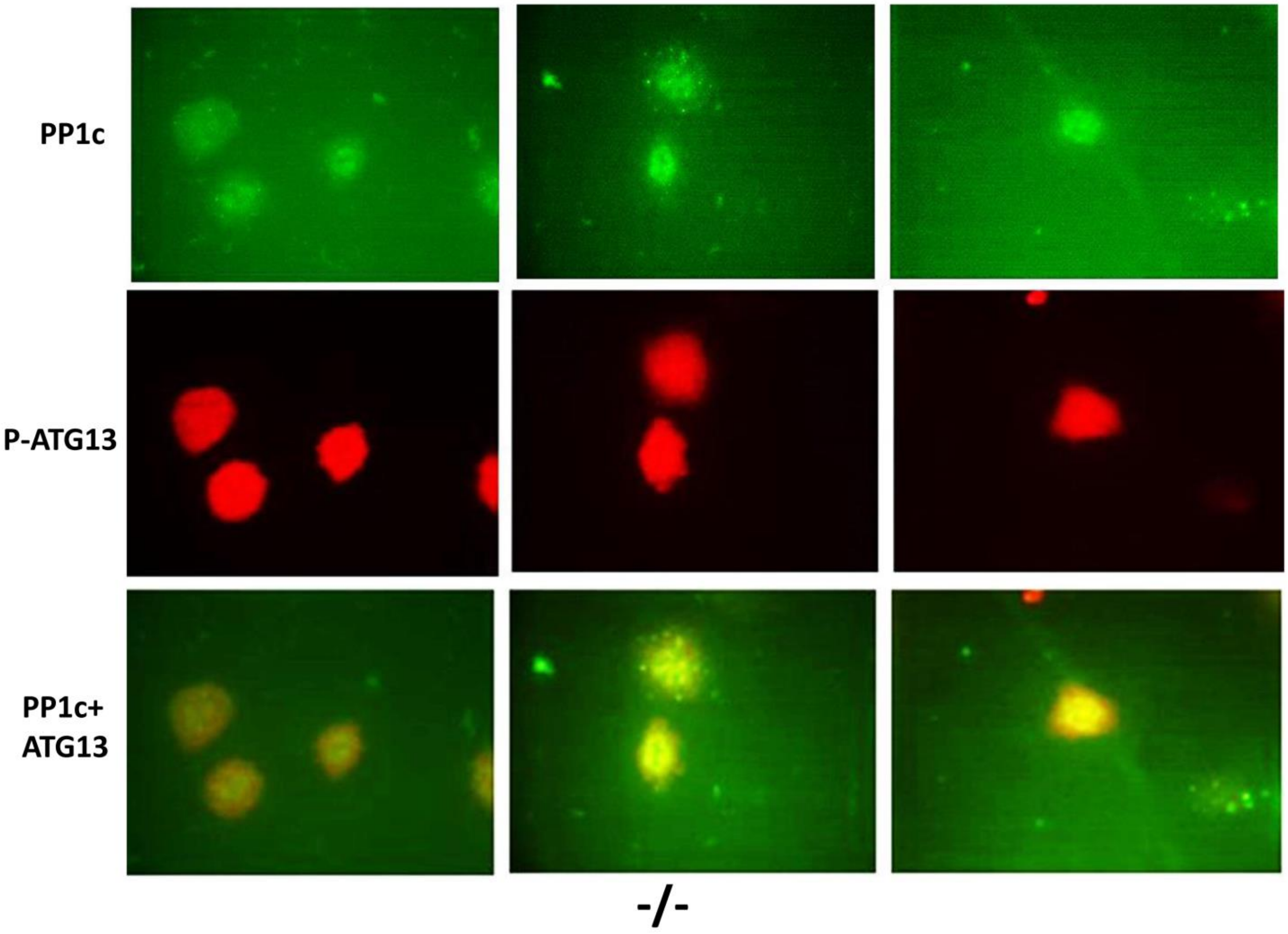

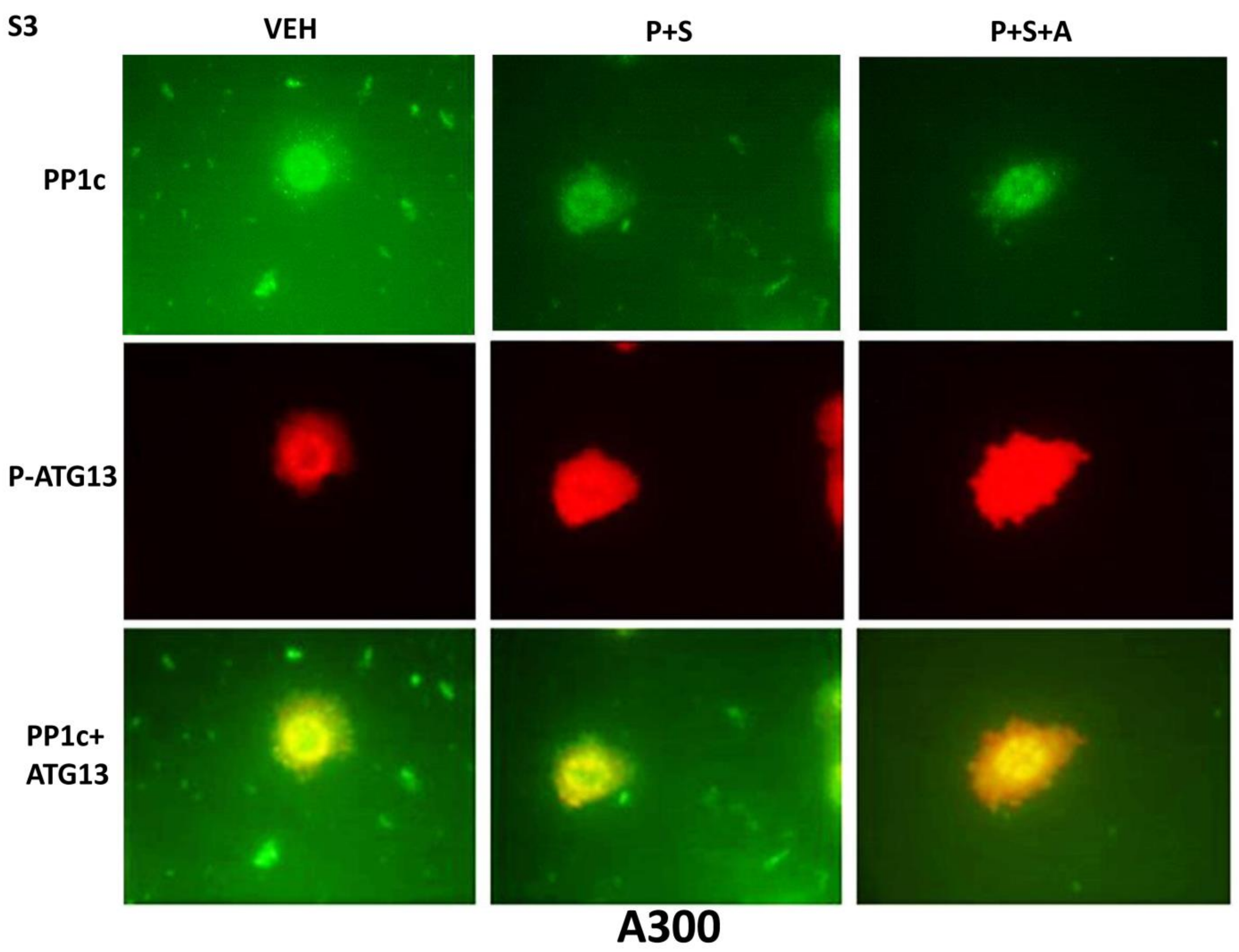

Figure S1; Figure S2; Figure S3. PP1c co-localization with ATG16L1 does not alter after drug exposure in ATG16L1 A300 A300 cells. HCT116 clones were treated with vehicle control, [pemetrexed (0.5 μM) and sorafenib (2 μM)], afatinib (0.5 μM) or the drugs in combination as indicated. Twelve h after treatment cells were fixed and permeabilized in situ and immuno-fluorescence was performed to determine the total phosphorylation / expression levels at 60X magnification of P-ATG S318 and PP1c; and the merged co-localization at 60X magnification of P-ATG13 S318 and PP1c.

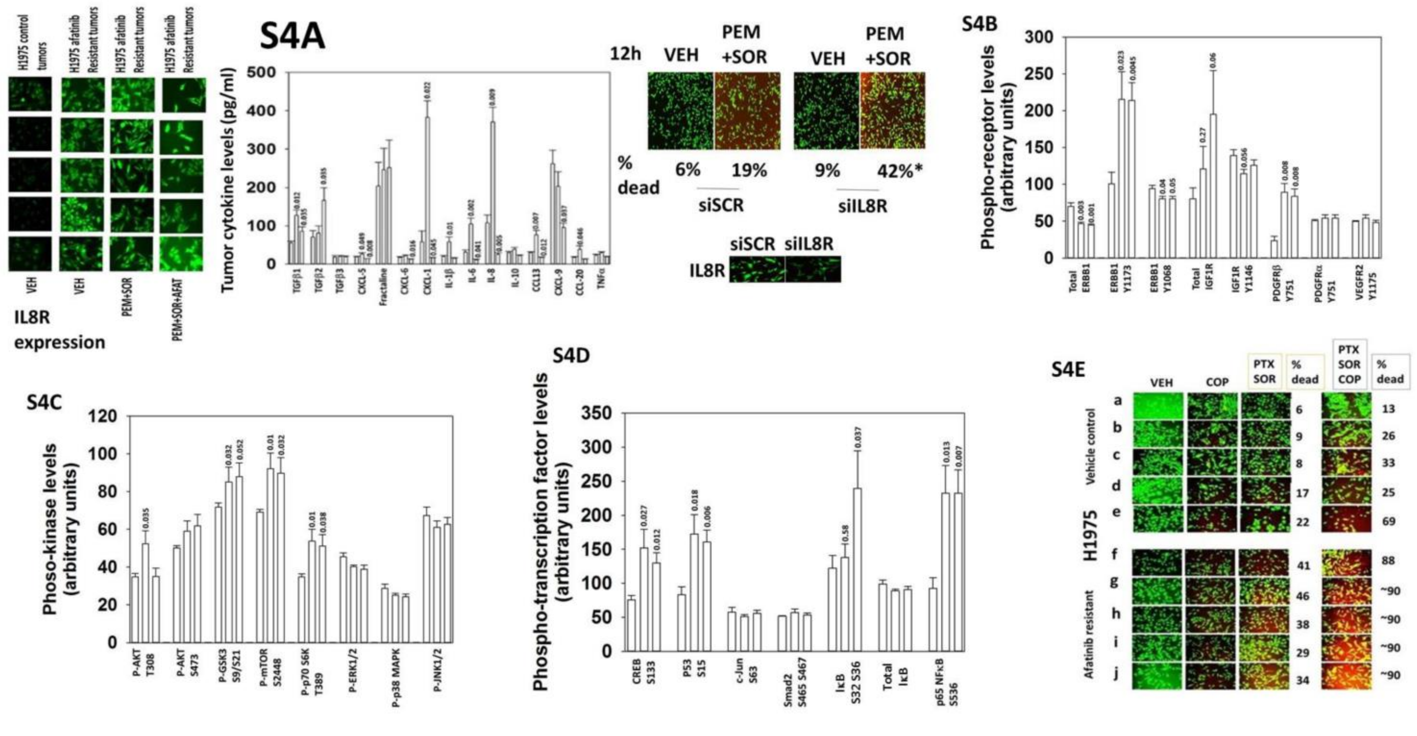
S4A
S4B
S4C
S4D
S4E
IL8R expression
H1975 control tumors
H1975 afatinib Resistant tumors
VEH
PEM+SOR
PEM+SOR+AFAT
Tumor cytokine levels (pg/ml)
12h
PEM +SOR
% dead
6%
19%
9%
42%*
siSCR
siIL8R
IL8R
Phospho-receptor levels (arbitrary units)
Phoso-kinase levels (arbitrary units)
Phospho-transcription factor levels (arbitrary units)
COP
PTX SOR
PTX SOR COP
Vehicle control
Afatinib resistant
H1975

Figure S4. [Pemetrexed + Sorafenib + Afatinib] treatment causes compensatory activation of PI3K and NFκB signaling. A.-D. Afatinib resistant H1975 tumors were generated as described in the Methods and 30 days after re-growth isolated and snap frozen. Tumor material from vehicle control and from [pemetrexed + sorafenib + afatinib] treated tumors was isolated ~30 days after drug exposure. Clarified tumor cell lysates were then subjected to multiplex assays as described in the Methods to detect the tumor levels of the indicated cytokines and phosphorylation status of signal transduction proteins using a Bio-Rad MAGPIX multiplex instrument (total 8 animals per condition, +/- SEM). *In Panels A-D the data are grouped in sets of three bars for each protein assessment, in order: vehicle treatment; afatinib resistant; [PTX+SOR+AFAT] treatment*. The data were analyzed using a two tailed Student's t test and the "*p* value" for each bar is placed above the bars. A *p* value of less than 0.05 is considered to be significant. **For Panel A. Left**: control and afatinib resistant tumor clones were treated with vehicle control, [pemetrexed (0.5 μM) + sorafenib (2 μM)], or [pemetrexed + sorafenib + afatinib (1.0 μM] for 6h. Cells were fixed and not permeabilized, and the expression of the cell surface IL-8 receptor determined. **Central portion**; multiplex graph showing afatinib resistant tumors over-express CXCL-1, IL-6 and IL-8. **Right** portion: Afatinib resistant clones were transfected with a scrambled siRNA or an siRNA to knock down the IL-8 receptor. Twenty four h after transfection cells were treated with vehicle control or with [pemetrexed (0.5 μM) + sorafenib (2 μM)]. Twelve h after drug treatment cells were treated with live / dead reagent and cells examined at 10X magnification using a Hermes WiScan microscope where red/yellow cells = dead; green cells = alive (n = 3 +/- SEM). **E.** Vehicle control H1975 clones and afatinib resistant H1975 clones were treated with vehicle control; with [copanlisib (0.5 μM)]; with [pemetrexed (0.5 μM) + sorafenib (2 μM)]; or with [pemetrexed (0.5 μM) + sorafenib (2 μM) + copanlisib (0.5 μM] for 24h. Twelve h after drug treatment cells were treated with live / dead reagent and cells examined at 10X magnification using a Hermes WiScan microscope where red/yellow cells = dead; green cells = alive.

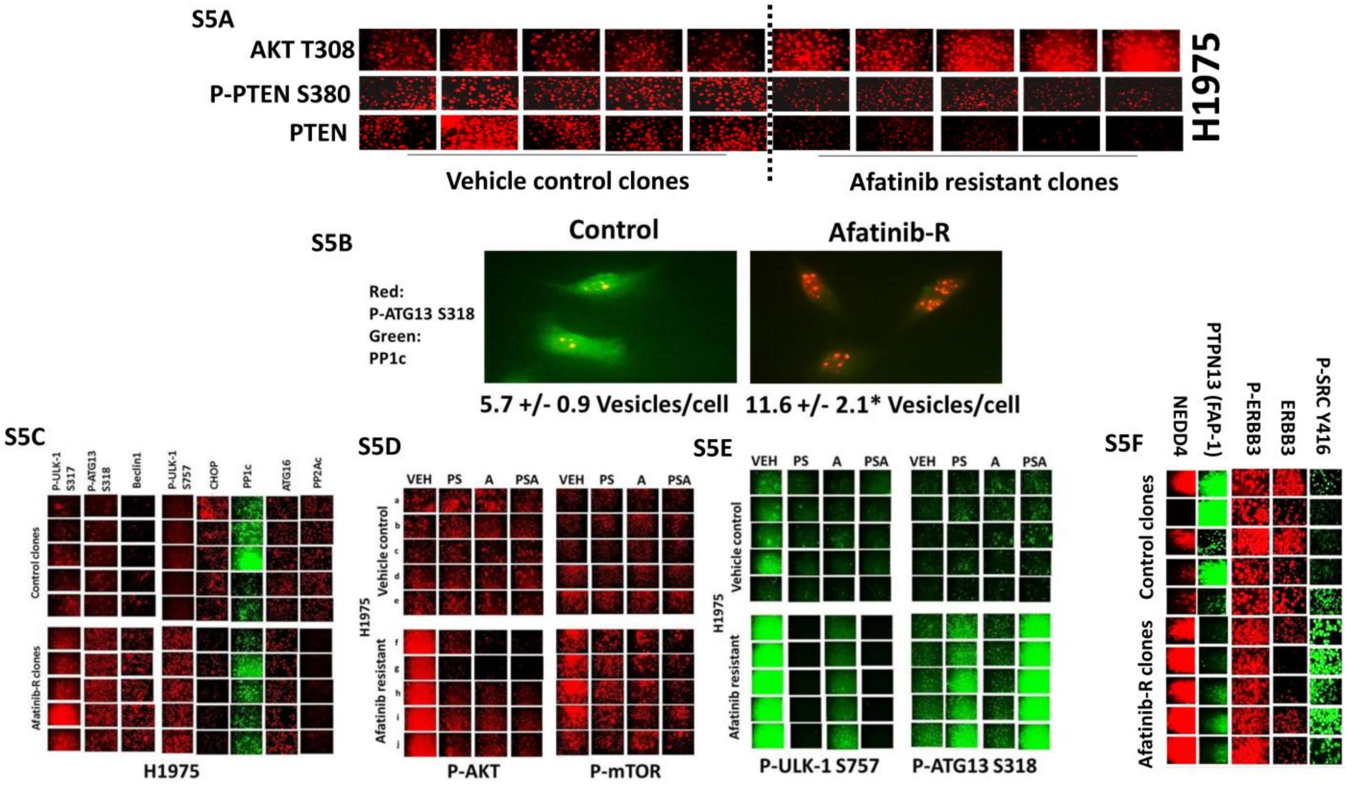

S5A
AKT T308
P-PTEN S380
PTEN
H1975
Vehicle control clones
Afatinib resistant clones
S5B
Control
Afatinib-R
Red:
P-ATG13 S318
Green:
PP1c
5.7 +/- 0.9 Vesicles/cell
11.6 +/- 2.1* Vesicles/cell
S5C
P-ULK-1 S317
P-ATG13 S318
Beclin1
P-ULK-1 S757
CHOP
PP1c
ATG16
PP2Ac
Control clones
Afatinib-R clones
H1975
S5D
VEH
PS
A
PSA
Vehicle control
Afatinib resistant
P-AKT
P-mTOR
S5E
P-ULK-1 S757
P-ATG13 S318
S5F
NEDD4
PTPN13 (FAP-1)
P-ERBB3
ERBB3
P-SRC Y416
Control clones
Afatinib-R clones

Figure S5. Clonal isolates of afatinib resistant H1975 tumors from in vivo passaging exhibit reduced expression of PTEN and a greater stoichiometry of PTEN S380 phosphorylation; afatinib resistant clones are more sensitive to [pemetrexed + sorafenib]. A. H1975 tumor clones (5 from vehicle control tumors; 5 tumors generated to become afatinib resistant) were dissociated and the clonal isolated cells grown in vitro as described in the Methods. Cells, 24h after plating in the absence of any drugs were fixed in situ and immuno-fluorescence was performed to determine the expression of PTEN and the phosphorylation status of AKT T308 and of PTEN S380. **B.** H1975 clones were fixed 24h after plating, permeabilized, and immuno-fluorescence performed to detect the levels of phospho-ATG13 S318 and of PP1c, and their co-localization (60X magnification). Afatinib resistant clones have a significantly greater number of ATG13 S318 foci than control clones and significantly less PP1c associated with those foci (* $p < 0.05$). **C.** H1975 tumors (5 from vehicle control; 5 generated to become afatinib resistant) were dissociated and the clonal isolated cells grown in vitro as described in the Methods. Cells, 24h after plating in the absence of any drugs were fixed in situ and immuno-fluorescence was performed to determine the expression of the indicated proteins and the phosphorylation of the indicated proteins. **D.-E.** H1975 clones were treated with vehicle control, afatinib (1 μM), [pemetrexed (0.5 μM) + sorafenib (2 μM)] or the three drugs combined for 6h. Cells were then fixed, permeabilized, and immuno-fluorescence performed to detect the levels of phospho-AKT T308, phospho-mTOR S2448, phospho-ULK-1 S757 and phospho-ATG13 S318 (10X magnification).

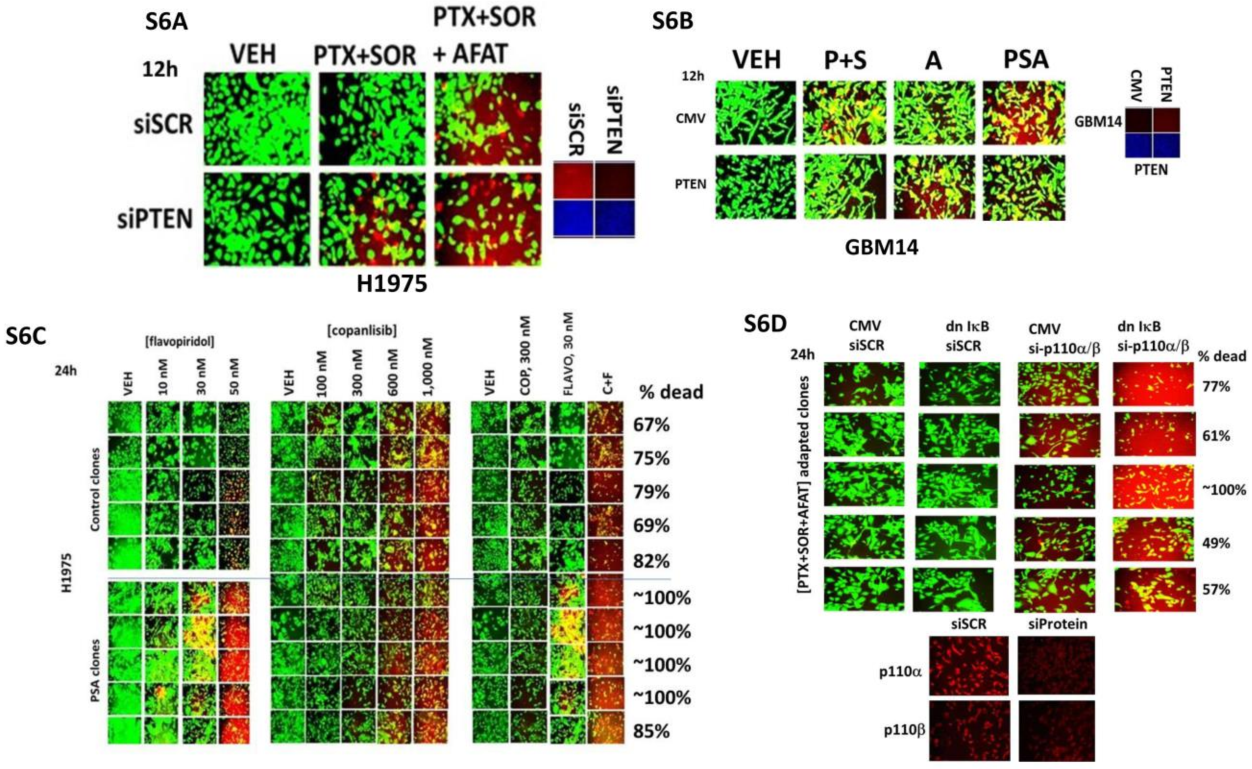
S6A
12h
VEH
PTX+SOR
PTX+SOR + AFAT
siSCR
siPTEN
siSCR
siPTEN
H1975
S6B
12h
VEH
P+S
A
PSA
CMV
PTEN
CMV
PTEN
GBM14
PTEN
GBM14
S6C
24h
[flavopiridol]
VEH
10 nM
30 nM
50 nM
[copanlisib]
VEH
100 nM
300 nM
600 nM
1,000 nM
VEH
COP, 300 nM
FLAVO, 30 nM
C+F
% dead
Control clones
PSA clones
H1975
67%
75%
79%
69%
82%
~100%
~100%
~100%
~100%
85%
S6D
24h
CMV siSCR
dn IκB siSCR
CMV si-p110α/β
dn IκB si-p110α/β
% dead
[PTX+SOR+AFAT] adapted clones
77%
61%
~100%
49%
57%
siSCR
siProtein
p110α
p110β

Figure S6. Loss of PTEN makes tumor cells more sensitive to [pemetrexed + sorafenib]. A. H1975 cells were transfected with a scrambled siRNA (siSCR) or an siRNA to knock down expression of PTEN. Twenty four h after transfection cells were treated with vehicle control; with [pemetrexed (0.5 μM) + sorafenib (2 μM)]; or with [pemetrexed (0.5 μM) + sorafenib (2 μM) + afatinib (1.0 μM] for 12h. Twelve h after drug treatment cells were treated with live / dead reagent and cells examined using a Hermes WiScan microscope where red/yellow cells = dead; green cells = alive. **B.** GBM14 (glioblastoma) were transfected with an empty vector plasmid (CMV) or a plasmid to express PTEN. Twenty four h after transfection cells were treated with vehicle control; with [pemetrexed (0.5 μM) + sorafenib (2 μM)]; or with [pemetrexed (0.5 μM) + sorafenib (2 μM) + afatinib (1.0 μM] for 12h. Twelve h after drug treatment cells were treated with live / dead reagent and cells examined using a Hermes WiScan microscope where red/yellow cells = dead; green cells = alive. **C.** Control H1975 clones and clones from H1975 tumors previously treated with [pemetrexed + sorafenib + afatinib] were treated with increasing concentrations of the IKK inhibitor flavopiridol (0-50 nM); increasing concentrations of the PI3K p110 catalytic activity inhibitor copanlisib (0-1,000 nM); or with flavopiridol (30 nM) and copanlisib (300 nM) combined. Twenty four h after drug exposure, cells were treated with live / dead reagent and cells examined using a Hermes WiScan microscope at 10X magnification where red/yellow cells = dead; green cells = alive. **D.** H1975 clones, previously treated with [pemetrexed + sorafenib + afatinib] were transfected with an empty vector plasmid (CMV) or a plasmid to express dominant negative IκB S32A S36A. In parallel, the same H1975 clones were also transfected with a non-specific scrambled siRNA (siSCR) or siRNA molecules to knock down the expression of PI3K p110α and PI3K p110β. Twenty four h after transfection cells were treated with live / dead reagent and cells examined using a Hermes WiScan microscope at 10X magnification where red/yellow cells = dead; green cells = alive.

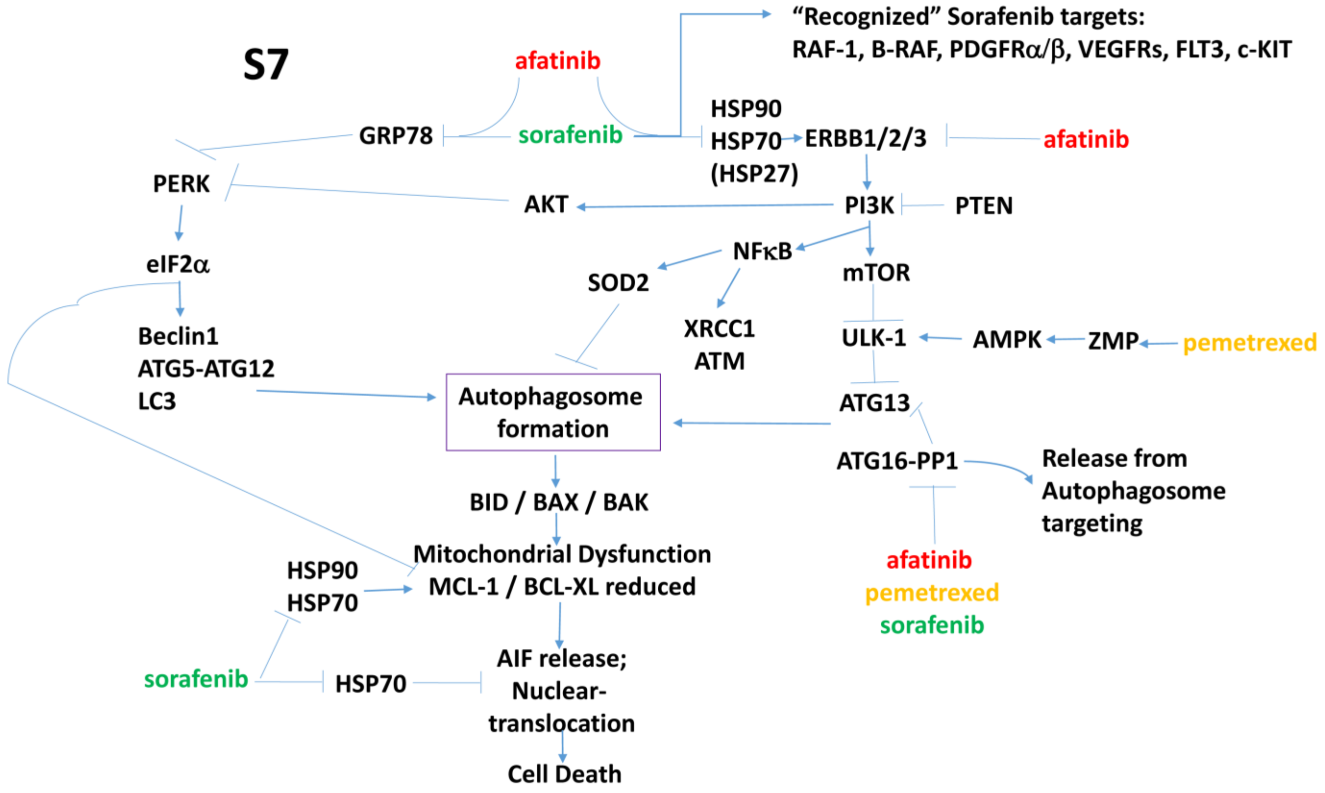

Figure S7. The molecular mechanisms by which pemetrexed, sorafenib and afatinib interact to kill tumor cells. Sorafenib can act as a weak inhibitor of chaperone ATPase inhibitor and it dysregulates HSP90 and HSP70 chaperones as well as altering the biology of small HSPs such as HSP27. This "sets the scene" for reduced signaling into the PI3K-(AKT)-mTOR pathway and increased signaling by PERK-eIF2α pathway that collectively through increased ATG13 S318 phosphorylation and elevated Beclin1 expression promote a toxic form of autophagy. Downstream of autolysosomes and the release of cathepsin and calpain enzymes into the cytosol is their cleavage of BID and with reduced expression of mitochondrial protective proteins leads to activation of BAX and BAK and the release of AIF into the cytosol. Because HSP70 function has been reduced AIF is more capable of translocating from the cytosol into the nucleus where it executes the tumor cell.
